# Knowledge of health workers on snakes and snakebite management and treatment seeking behavior of snakebite victims in Bhutan

**DOI:** 10.1371/journal.pntd.0008793

**Published:** 2020-11-30

**Authors:** Sunil Sapkota, Deb P. Pandey, Guru P. Dhakal, Dhan B. Gurung

**Affiliations:** 1 Department of Forest Science, College of Natural Resources, Lobesa, Punakha, Bhutan; 2 Raise Hands Nepal, Bharatpur-15, Chitwan, Nepal; 3 Department of Veterinary Microbiology and Parasitology, Agriculture and Forestry University, Bharatpur, Chitwan, Nepal; 4 School of Medicine and Public Health, Faculty of Health and Medicine, University of Newcastle, Callaghan, NSW 2308, Australia; 5 Jigme Dorji Wangchuck National Referral Hospital, Thimphu, Bhutan; College of Health Sciences, Bayero University Kano, NIGERIA

## Abstract

**Background:**

Published information on snakebite is rare in Bhutan although remarkably higher number of snakebites and associated deaths are reported from other South Asian countries.

**Aims and methodology:**

Structured questionnaire was used to collect knowledge of health workers in snakebite management and health seeking behavior of snakebite victims as observed by health workers. Study was conducted in purposively sampled 10 Dzongkhags (district level administrative units) with higher incidence of snakebites.

**Result:**

Heath workers scored 27–91% (with an average of 63%, *SD* = 14) for 52 questions related to snake identification and snakebite management. Among 118 health workers interviewed, 23% had adequate knowledge on snakes and snakebite management while 77% had inadequate knowledge. Among 32 Doctors, 63% of them scored above or equal to 75%. Health workers from Sarpang scored higher (76%, *SD* = 11) than those from other Dzongkhags. Snakebite victim's visit to local (traditional) healers prior to seeking medical help from hospital was observed by 75 (63%) health workers. Fifty one percent of health workers observed patients treated with local methods such as the use of black stone called *Jhhar Mauro* (believed to absorb snake venom), application of honey, rubbing of green herbal paste made up of *Khenpa Shing* (*Artemisia myriantha* Wallich ex Besser var. paleocephala [Pamp] Ling) and consumption of fluid made up of Neem leaf (*Azadirachta indica* Juss). Use of tight tourniquet as a first aid to snakebite was observed by 80% of the health workers.

**Conclusion:**

Health workers lack confidence in snakebite management. Snakebite victims are likely to suffer from harmful local practices and traditional beliefs on local treatment practices. Empowering health workers with adequate knowledge on snakebite management and making locals aware in pre-hospital care of snakebites are needed to improve the pre- and in-hospital management of snakebite in Bhutan.

## Author summary

Snakebites affect people globally including Bhutan and snakes are source of fear and excitement. Major proportion (62.7%) of Bhutanese population who thrive on agro-farming, rearing livestock, and searching forest products are at risk of snake envenomations. Deaths due to snakebites are dependent upon use of first aid type, provisions available with the health facilities, and health seeking behaviors of snakebite victims. This multi-center study was conducted to assess the level of knowledge of health workers and its association with their demographic features and health seeking behavior of snakebite victims as observed by health workers using investigator administered structured questionnaire. We included 12 hospitals and a Basic Health Unit (BHU) from 10 snakebite prone Dzongkhags with 962 (84%) snakebites from January 2013 to December 2017 in the country. Among 118 health workers interviewed, 27 (23%) had adequate level of knowledge with remaining 91 (77%) having inadequate knowledge. Male health workers and Doctors had better level of knowledge than other study groups. Health workers from Sarpang scored higher compared to those from other Dzongkhags. A total of 42% (*n* = 50) health workers scored below 60% (31 or less correct responses for 52 questions), which demands requirement of training and inclusion of practice-oriented courses in training curriculum of health professionals for snakebite management. Often, use of traditional medication and less frequent adherence to WHO recommended first aid measures was observed by 5% (*n* = 6) respondents. The adoption of non-recommended treatment practices seems to delay proper treatment resulting in gangrene and associated complications.

## Introduction

Rural, agro-based livelihood of Asian and African community contributes to increased snake–human interactions [1]. World Health Organization (WHO) Guidelines [2] define snakebite as an environmental and occupational disease. Retrospective studies [3,4] from Bhutan supports the statement. Maintenance of the health of farmers and their families often termed "rural health" has assumed increasing importance in most South Asian countries. The sustainable development goal number two (basic health rights for all) cannot be achieved without ensuring the health and safety of people associated with snakebite risks. In Bhutan, 62.7% of the population depends on self-subsistent agriculture [5]. The country has 70.1% of its land area under forest cover [6]. It houses 69 species of snakes representing five families (Colubridae, Elapidae, Pythonidae, Typhlopidae, and Viperidae), nine neurotoxic elapids (*Bungarus bungaroides* Cantor, *B. caeruleus* Schneider, *B. fasciatus* Schneider, *B. lividus* Cantor, *B. niger* Wall, *Naja kaouthia* Lesson, *N. naja* Linnaeus, *Ophiophagus hannah* Cantor, and *Sinomicrurus macclellandii* Reinhradt [7–9]), and eight hemotoxic vipers (*Daboia russelii* Shaw and Nodder, *Gloydius himalayanus* Gunther, *Ovophis monticola* Gunther, *Protobothrops himalayanus* Pan, Chettri, Yang, Jiang, Wang, Zhang and Vogel, *Protobothrops jerdonii* Gunther, *Trimeresurus albolabris* Gray, *T. erythrurus* Cantor, and *T. popeiorum* Smith [7,9,10]). Genera *Boiga*, *Chrysopelea* and *Rhabdophis* in Colubridae family have potential to cause health complications following their bites [11]. Published source reported that Bhutan use Indian polyspecific antivenom for management of snakebites [4].

In Bhutan allopathic and indigenous government-financed medical systems provide free healthcare facilities in 211 Basic Health Units (BHU), 52 sub-posts, 551 Outreach Clinics (ORCs) at primary level, 26 hospitals at secondary level, three referral hospitals at the tertiary level, and 61 Traditional Medicine units and one national Traditional Medicine hospital. There are 345 clinicians providing allopathic medicines and 55 indigenous physicians ("*Drungtshos*") providing indigenous medical facilities spread across the country [12].

A retrospective study from the Central Regional Referral Hospital in Gelephu, Sarpang gives insight about demographic features of snakebite victims from 2013–2016 [4]. Based on the medical registers, the annual health bulletin from Bhutan [3] summarizes annual snakebites in between 2013 to 2018. This summary results an average of 221 snakebites and one snakebite caused death annually in Bhutan [3].

Time to access hospital care and anti-snake venom serum (ASVS) administration after bite play a vital role in saving the life of a snakebite victim [2,13–17]. Competence of the health workers (Doctors, Nurses, and Emergency Medical Technician) to correctly identify venomous snake, the features of venomous bites, administration of appropriate first aid measures, ASVS administration in medical setup and airway or ventilation are essential in the survival of envenomed cases [16–18].

The behavior of rural Bhutanese communities in managing the snakebite cases following snakebites and existence of snakebite treatments through local methods was still unknown. Presence and practices of traditional healers who provide *Tantrik* and herbal medications in remote areas of Bhutan have not been documented till date. Reciting of *mantras*, use of herbal medicines, and other practices by rural people despite having access to medical facilities seem unabated. Such practices need scientific validation in reducing snakebite related health risks. Lack of literature or ignorance on snake and snakebite management practices are barriers to reduce snakebite mortality [16]. Literature on snakebite management practices in Bhutan are scarce. Therefore, this study aims to report knowledge of medical personnel on snakebite management including snake identification and behavior of snakebite victims in snakebite management in Bhutan.

## Methods

We conducted multi-center study to access level of knowledge of health workers using structured questionnaire (S1 File). Three surveyors were employed for data collection, two trained surveyors; one each for Samtse and Sarpang were hired to administer the question and record the responses. Principal investigator (PI) administered and recorded responses from other eight dzongkhags. Survey was conducted in English as the respondents were literate and passed their course studying in English medium school. English is one of the official languages used for administrative communication in Bhutan. Questions were related to key features of identifying common venomous snakes reported in Bhutan, use of first aid for snakebite, laboratory test for snakebite diagnosis, signs and symptoms of snakebite and probable complications after injecting ASVS. Association of their knowledge with demographic features was evaluated. Health seeking behavior of snakebite victims as observed by health workers using structured questionnaire were also recorded.

Health workers were asked to decide on displayed snake as venomous or non-venomous based on visual appearance and physical position in photograph. Most of the health workers decided the nature of snakes based on appearance and size in photographs. They were also inquired about the conditions of snakebite victims who reported to the hospitals.

**Fig 1:**
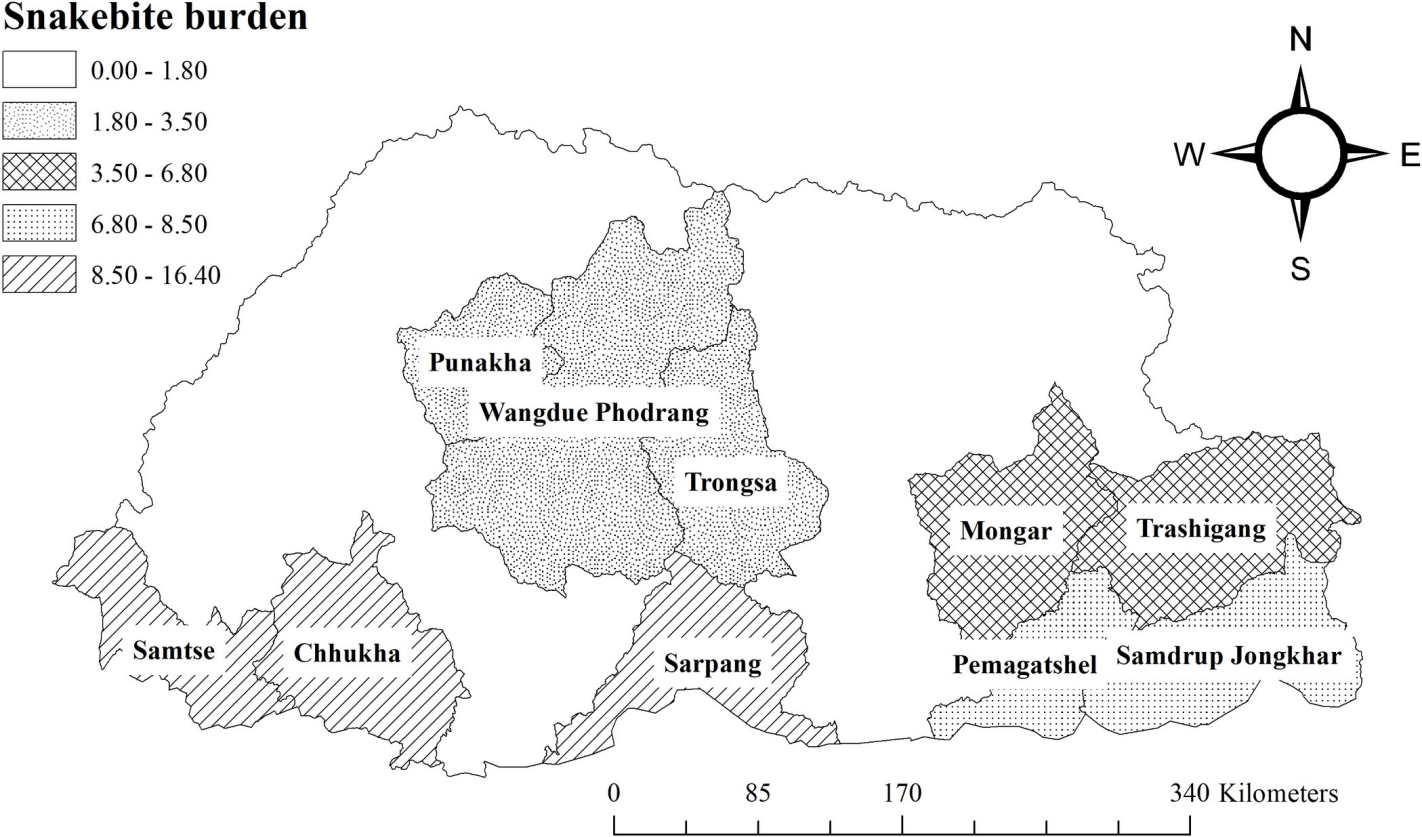
Study Dzongkhags with average annual percent of snakebite cases reported in each Dzongkhag per year in Bhutan.

We purposively included 12 district hospitals (from a total of 20 districts, including the central and eastern referral hospital), a general hospitals, and a Basic Health Unit (BHU) from 10 snakebite prone Dzongkhags (Samtse, Chhukha, Sarpang, Wangdue Phodrang, Punakha, Trongsa, Mongar, Trashigang, Pemagatshel and Samdrup Jongkhar). These district hospitals reported 962 (84% of total snakebite burden) snakebite cases from January 2013 to December 2017 in the country (Fig 1, S1 Table). Dzongkhag hospitals were included as snakebite cases are primarily referred there from BHUs. Further, complicated cases are referred to the Regional Referral and National Referral Hospitals.

### Study design

We interviewed all health workers involved in In-Patient Ward (IPD) and Emergency Ward of a BHU, Dzongkhags, General and Referral hospitals. We administered questionnaire to assess the knowledge of health workers based on their experience of caring snakebite patient at hospitals. Recently published snake fauna diversity in Bhutan [7,9], a study from Bangladesh [19], relevant publications [15, 17], and WHO guidelines for the treatment of snakebites [1,13] were referred to prepare the questions. Health workers were enquired for their on duty observations of any local or traditional treatment sought by snakebite victims prior to visiting the hospital.

Each question had two to four distractors and a correct option. A score of 2 was given to every correct and zero to incorrect response. For questions on signs and symptoms, laboratory tests, and complication after injecting anti-snake venom to snakebite victims; a score of 2 for correct response, 1 for acceptance on provocation by interviewer and zero for rejection or no idea were awarded. The question also focused on the observation made by the health workers on the local/traditional treatment sought by the snakebite victims before reaching hospital in addition to the option such as the use of pressure bandage and immobilization of limb with splint.

### Statistical method

Scores for individuals were recorded and summed in MS Excel spreadsheets. The resulting sum ranging 75%-100% were interpreted as health workers having adequate knowledge and those with less than 75% as inadequate knowledge. Association between knowledge and socio-demographic features of health workers were analyzed using IBM SPSS trial version.

Observational response of health seeking behavior of snakebite patients were reported with the cumulative frequency. WHO recommended first aid, visiting local healers for ethno-medical help, and other traditional practices were considered responses for treatment seeking behavior. Responses in agreement to WHO guidelines were given the score of 1 and the rest 0. The frequencies were summed.

### Inclusion and exclusion criteria

We selected nurses and medical staff engaged in emergency department of sampled health institutions. The medical staffs not available at the time of the interview and not willing to participate were excluded from the study. We excluded those who were not supposed to be involved in snakebite treatments.

### Ethical consideration

We obtained institutional approval from Policy and Planning Division (PPD/ADM.CL/9/2018-19) and ethical approval from Research Ethics Board of Health (REBH) (REBH/Approval/2018/107) under Ministry of Health, Thimphu, Bhutan. We informed participants and assured anonymity in any publication. For the voluntary participation of the respondents, we provided non-monetary incentives such as educational trainings about identification of venomous snakes using photographs and brochure and pamphlets on ‘Medically Important Snakes of Bhutan’. We obtained signed informed consent prior to including respondents in this study (S2 File).

## Results

Scores for knowledge on snakes and snakebites among health workers in our study sites in Bhutan ranged 27–93% (mean (*m*) = 63%, standard deviation (*SD*) = 14%). Among 118 health workers interviewed, 29 (25%) were found to have adequate knowledge while the remaining 89 (75%) had inadequate knowledge.

### Age and experience of health workers

Age of interviewed health workers ranged from 24 to 55 years (*m* = 32, *SD =* 8). Their professional experience ranged from 1 to 37 years (*m* = 8, *SD =* 8). Respondents stayed for an average of 5 years (*SD* = 4) in the current place of posting. A respondent from Sarpang managed 100 snakebites, while 19% did not manage any snakebite case. The average number of snakebite cases managed by health workers was 9 (*SD* = 13) (Table 1).

**Table 1:** Age, professional experience, and snakebite case managed by health worker.

	*m*	*SD*	*SE*
Age in years	31.90	7.49	0.690
Professional experience in years	7.47	7.99	0.736
Years served in rural areas	1.45	4.12	0.379
Years served in urban areas	6.24	6.83	0.629
Number of snakebites managed	9.10	13.16	1.211
Years of stay in current place of posting	4.56	4.35	0.401

*m* = Mean, *SD* = Standard deviation, *SE* = Standard error.

### Source of knowledge

Regarding source of basic knowledge for snake identification and snakebite management among 118 health workers, 108 (92%) learnt it from curricular books during their professional training and 2 (2%) received training specifically on snake identification and snakebite management. Seventy seven (62%) respondents acquired knowledge through internet, watching television and listening to radio programs while 20 (17%) said that folk tales and talks in their families and villages helped them on snake identification and snakebite management.

### Confidence in snakebite management

Majority (55%, *n* = 65) of the health workers expressed the lack of confidence or having no confidence or no idea on snake identification. However, 75% (*n* = 89) of the participants had confidence on how to prevent snakebite. On the other hand, 90% (*n* = 107) of the health workers were very confident or moderately confident on administering first aid to snakebite cases and interpreting signs and symptoms of snakebites. About 15% (*n* = 18) health workers were not confident about probable complications after injecting ASVS. On the need for laboratory tests required for snakebite management, 76% (*n* = 90) of the participants expressed confidence. For all six variables of snakebite management, respondents expressed moderate confidence (Table 2). Health workers expressed lack of confidence in snake identification skills.

**Table 2:** Confidence of health workers on different variables.

Confidence on	VC(%)	MC(%)	LC(%)	NC(%)	NI(%)
Identification of venomous snakes	3(2.5)	50(42.4)	28(23.7)	27(22.9)	10(8.5)
Preventive measures of snakebite	26(22)	63(53.4)	15(12.7)	9(7.6)	5(4.2)
First aids for snakebite	49(41.5)	58(49.2)	8(6.8)	1(0.8)	2(1.7)
Signs and symptoms of snakebite	49(41.5)	58(49.2)	11(9.3)	0	0
Probable complications after injecting ASVS	39(33.1)	42(35.6)	21(17.8)	5(4.2)	11(9.3)
Lab. tests for snakebite	35(29.7)	55(46.6)	15(12.7)	7(5.9)	6(5.1)

VC = Very Confident, MC = Moderately Confident, LC = Lack Confidence, NC = No Confidence, NI = No Idea, % in parentheses indicates percentage of frequency of abbreviated response.

All health workers stressed the need for training on snake identification and snakebite management. Among 118 respondents, 86% (*n* = 101) said that the training was very important, 9% (*n* = 11) said it is of moderate importance, and remaining 5% (*n* = 6) said it is important.

### Identification of snakes

Among 118 respondents, 88% (*n* = 104) and 85% (*n* = 100) correctly identified photographs of Common Cobra (*Naja naja*) and Monocled Cobra (*Naja kaouthia*) as venomous. Table 3 shows snake identification ability from among the displayed snake photographs.

**Table 3:** Snake identification using photographs.

Snake shown in photo	V	V%	NV	NV%	NI	NI%
*Boiga multifasciata* (S1 Fig)	81*	68.6	9	7.6	28	23.7
*Ptyas mucosa* (S2 Fig)	35	29.7	36*	30.5	47	39.8
*Bungarus fasciatus* (S3 Fig)	50*	42.4	22	18.6	46	39.0
*Daboia russelii* (S4 Fig)	56*	47.5	27	22.9	35	29.7
*Trimeresurus albolabris* (S5 Fig)	70*	59.3	28	23.7	20	16.9
*Amphiesma stolatum* (S6 Fig)	55	46.6	19*	16.1	44	37.3
*Amphiesma platyceps* (S7 Fig)	40	33.9	27*	22.9	51	43.2
*Python molurus bivittatus* (S8 Fig)	44	37.3	48*	40.7	26	22.0
*Bangarus niger* (S9 Fig)	20*	16.9	43	36.4	55	46.6
*Ptyas nigromarginata* (S10 Fig)	43	36.4	42*	35.6	33	28.0
*Naja kaouthia* (S11 Fig)	100*	84.7	3	2.5	15	12.7
*Xenochrophis piscator* (S12 Fig)	51	43.2	22*	18.6	45	38.1
*Oligodon juglandifer* (S13 Fig)	41	34.7	27*	22.9	50	42.4
*Rhabdophis subminiatus* (S14 Fig)	41*	34.7	24	20.3	53	44.9
*Pseudoxenodon macrops* (S15 Fig)	28*	23.7	36	30.5	54	45.8
*Ovophis monticola* (S16 Fig)	56*	47.5	22	18.6	40	33.9
*Naja naja* (S17 Fig)	104*	88.1	0	0.0	9	7.6
*Lycodon aulicus* (S18 Fig)	52	44.1	19*	16.1	47	39.8
*Ophiophagus hannah* (S19 Fig)	75*	63.6	14	11.9	29	24.6
*Bangarus lividus* (S20 Fig)	37*	31.4	38	32.2	43	36.4

*indicates the correct option; snake species are arranged in the sequence they were displayed to participants (V = indicates the response venomous, NV = indicates the response non-venomous NI = indicates the response No idea and respective abbreviation with % indicates percentage of abbreviated response).

On question to key identification features of Common Cobra, majority (56%, *n* = 66) selected spectacle mark present behind the hood. Eighty four percent of the respondents did not know the presence of large hexagonal mid-dorsal scales as the basic identification feature of kraits (Table 4). In contrast, 55% (*n* = 65) said triangular head with irregular scales as identification key for vipers. Also, 59% (*n* = 70) said the terms venomous and poisonous are similar in meaning.

**Table 4:** Identification features of venomous snake species.

Question	Answers	Count	%
Which of the following key features of snake help you to identify Common Cobra?	Shiny color	11	9.3
	Paired white bands along the body	13	11.0
	Black head with brown body	13	11.0
	Spectacle present behind the hood	66^a^	55.9
	No idea	15	12.7
Which of the following key features of snake help you to identify Kraits?	Paired white bands along the body	24	20.3
	Large hexagonal mid-dorsal scales	20^a^	16.9
	Black head with brown body	16	13.6
	Spectacle present behind the hood	4	3.4
	No idea	54	45.8
Which of the following key features of snake help you to identify Vipers?	Triangular head with irregular scales	65^a^	55.1
	Large hexagonal mid-dorsal scales	5	4.2
	Black head with brown body	7	5.9
	Spectacle present behind the hood	3	2.5
	No idea	38	32.2
Venomous and poisonous are similar	Yes	69	58.5
	No	38^a^	32.2
	No idea	11	9.3

^a^ indicates the correct option, *%* = Percentage among total.

### Preventive measures of snakebite

On the strategies required to prevent snakebite, 82% (*n* = 97) respondents mentioned cleaning and clearing bushes and debris, 53% (*n* = 62) mentioned use of protective equipment, 53 (45%) mentioned avoiding marshy and busy area, and 38% (*n* = 45) mentioned covering the holes in the surrounding. Among 118, 53% (*n* = 63), 41% (*n* = 48), 65% (*n* = 78) and 42% (*n* = 50) respectively believed spraying phenol, kerosene, alcohol, and garlic syrup expecting to ward off snakes. Also, 78% (*n* = 92) of the health workers did not believe on praying to local deities or god to reduce snakebites. While 12% (*n* = 14) participants accepted and 86% (*n* = 101) rejected if killing snakes could prevent future snakebite, 3% (*n* = 3) said that they do not have any idea if hunting and killing snakes can help to prevent snakebite (Table 5).

**Table 5:** Methods to prevent snakebites.

Preventive measures of snakebite	M	M%	AP	AP%	RI	RI%	NI	NI%
Spraying phenol^!^	19	16.1	44	37.3	24	20.3	31	26.3
Hunting and killing snakes^!^	0	0.0	14	11.9	101	85.6	3	2.5
Cleaning and clearing bushes, and debris laying on the ground*	97	82.2	21	17.8	0	0.0	0	0.0
Spraying kerosene^!^	11	9.3	37	31.4	38	32.2	32	27.1
Use of protective equipment*	62	52.5	55	46.6	0	0.0	1	0.8
Cover up holes in surrounding*	45	38.1	60	50.8	9	7.6	4	3.4
Avoid marshy and busy area*	53	44.9	59	50.0	3	2.5	3	2.5
Spraying alcohol^!^	30	25.4	47	39.8	23	19.5	18	15.3
Spraying garlic syrup^!^	17	14.4	33	28.0	20	16.9	48	40.7
Praying to local deities/god^!^	3	2.5	9	7.6	94	79.7	12	10.2

* = Recommended methods to reduce snakebite

^!^ = Non-recommended due to unproven/doubtful benefits or no benefits at all, M = Mentioned, AP = Accepted when provoked, RI = Rejected the idea, NI = No idea and respective abbreviation with % indicates percentage of abbreviated response.

### First aid for snakebite

When asked for appropriate site for tourniquet, 72% (*n* = 85) of the respondents said it should not be used. Seventy one percent respondents said that snakebite mark should not be covered. On an arrival of victim to hospital, 92% (*n* = 109) of participants said that they reassured and calmed the patients. About 68% (*n* = 80) of health workers said that reassuring and calming of the snakebite victim prevents complication and helps in observing signs and symptoms. The best suggestion to manage future snakebite properly from 70% (*n* = 83) of the respondents was carrying victim in a comfortable transport to the nearest hospital provided with ASVS immediately (Table 6).

**Table 6:** First aid of snakebite.

Question	Answers	Count	%
Appropriate site for tourniquet is	Over the site of bite	3	2.5
	2 inches above the site of bite	29	24.6
	2 inches below the site of bite	0	0.0
	Should not be used	85^a^	72.0
	No idea	1	0.8
Bite mark should be covered with bandage	Yes	29	24.6
	No	84^a^	71.2
	No idea	5	4.2
When a snakebite victim reports to hospital you will	Apply pressure bandage	6	5.1
	Slice the wound	1	0.8
	Suck the wound	2	1.7
	Reassure and calm the patient	109^a^	92.4
	No idea	0	0
Reassuring and calming of the snakebite victim helps to	Prevent complication and helps in observing sign and symptom	80^a^	67.8
	Reduces bleeding	1	0.8
	Reduces rate of venom diffusion	34	28.8
	Induces sleep	1	0.8
	No idea	2	1.7
Best suggestion to manage future snakebite properly is to	Find local healers	3	2.5
	Quickly take victim in a comfortable transport to nearest hospital provided with ASVS	82^a^	69.5
	Find the snake and kill it for identification	3	2.5
	Go to the nearest clinic	30	25.4
	No idea	0	0

^a^ indicates the correct option, % = Percentage among total.

### Signs and symptoms of snakebite

Majority of the respondents mentioned swelling of wound, pain and blisters (95%, *n* = 112), fang marks (96%, *n* = 113), low blood pressure / high pulse rate (98%, *n* = 116), and hematuria / bruises / red marks around wound (99%, *n* = 117) as signs and symptoms of snakebites (Table 7). Hematuria and swelling are general symptoms of hemotoxic bites mentioned by participant health workers.

**Table 7:** Signs and symptoms of snakebite envenomations.

Signs and Symptoms	M	M%	AP	AP%	RI	RI%	SI	SI%
Swelling wound, pain and blisters	112	94.9	4	3.4	2	1.7	0	0
Dizziness and vomiting	56	47.5	50	42.4	4	3.4	8	6.8
Blurring of vision	34	28.8	72	61.0	3	2.5	9	7.6
Convulsion	15	12.7	71	60.2	6	5.1	26	22
Unconsciousness/mental confusion	54	45.8	57	48.3	2	1.7	5	4.2
Dropping of eyelids/ptosis	58	49.2	44	37.3	2	1.7	14	12
Weakness of neck muscle	36	30.5	64	54.2	1	0.8	17	14
Difficulty in swallowing	26	22.0	67	56.8	5	4.2	19	16
Nasal regurgitation/voice	22	18.6	43	36.4	7	5.9	46	39
Difficulty in respiration	70	59.3	43	36.4	0	0	5	4.2
Bleeding from gum and vomiting	57	48.3	42	35.6	6	5.1	13	11
Persistent bleeding from bite site	60	50.8	35	29.7	12	10	11	9.3
Severe muscle pain	50	42.4	57	48.3	2	1.7	9	7.6
Dark colored urine	33	28.0	52	44.1	2	1.7	31	26
Scanty or no urine output	31	26.3	59	50.0	5	4.2	23	19
Renal failure	43	36.4	61	51.7	0	0	14	12
Shock/collapse	48	40.7	63	53.4	0	0	7	5.9
Fang marks	113	95.8	5	4.2	0	0	0	0
Low blood pressure / high pulse rate	116	98.3	2	1.7	0	0	0	0
Hematuria / bruises / red marks around wound	117	99.2	1	0.8	0	0	0	0

M = Mentioned, AP = Accepted when provoked, RI = Rejected the idea, NI = No idea and respective abbreviation with % indicates percentage of abbreviated response.

Among all, 64% (*n* = 76) of health workers said that cobra venom mainly causes neurotoxic effect. Only 40% (*n* = 47) of the health workers knew viper venom causes hemotoxic effect. Forty percentage of interviewed health workers said that tissue death at bitten site is generally caused by cobra bite. Seventy six percentage (*n* = 90) of health workers said that drooping of eyelids is important symptoms of neurotoxic envenomation. While, 54% (*n* = 64) of them were aware about the fact that respiratory failure is mostly caused by krait bite. If no envenomation effects developed during observation period at hospital conditions, 74% (*n* = 87) of respondents declared patient was not envenomed. Polyvalent ASVS administration was recommended after development of envenomation symptoms by 73% (*n* = 86) of the respondents (Table 8).

**Table 8:** Idea on signs and symptoms of snakebite.

Question	Answers	Count	%
Cobra venom mainly causes	Nephrotoxic effect	11	9.3
	Neurotoxic effect	76^a^	64.4
	Cytotoxic effect	0	0.0
	Hemotoxic effect	17	14.4
	No idea	14	11.9
Viper snakebite mainly causes	Nephrotoxic effect	12	10.2
	Neurotoxic effect	23	19.5
	Cytotoxic effect	8	6.8
	Hemotoxic effect	47^a^	39.8
	No idea	28	23.7
How can you declare non-venomous snakebites if a snakebite victim arrive in your center?	Swelling, pain on bitten site	3	2.5
	Wound with many dots	15	12.7
	Drooping eyes with scratch/two dots and broken neck symptoms	6	5.1
	After the observation if no symptoms occur	87^a^	73.7
	No idea	7	5.9
Tissue death at bitten site is generally caused by	Cobra bite	47^a^	39.8
	Wolf snake bite	2	1.7
	Rat snake bite	8	6.8
	Krait bite	21	17.8
	No idea	40	33.9
Respiratory failure is mostly caused by	Krait bite	64^a^	54.2
	Rat snake bite	5	4.2
	Python bite	12	10.2
	Wolf snake bite	2	1.7
	No idea	35	29.7
Important symptoms of neurotoxic envenomation is	Vomiting	9	7.6
	Nausea	5	4.2
	Drooping of eyelids	90^a^	76.3
	Body pain	2	1.7
	No idea	12	10.2
Polyvalent ASVS can be administered for	When patients develop symptoms of envenomation	86^a^	72.9
	Snakebite patient has fang marks	16	13.6
	After all snakebite	3	2.5
	Snakebite patients is in fear and unconsciousness	2	1.7
	No idea	11	9.3

^a^ indicates the correct option, % = Percentage among total.

### Lab tests for snakebite management

Majority of the respondents mentioned 20 minutes whole blood clotting (89%, *n* = 105), complete blood count (83%, *n* = 98) and bleeding time/clotting time (97%, *n* = 114) as lab test to decide on snakebite envenomation. Blood urea test or creatinine and electrolyte test, renal function test were mentioned by 78% (*n* = 92) of the health workers (Table 9).

**Table 9:** Responses on a query about lab test applicable for snakebites.

Knowledge on lab test	M	M%	AP	AP%	RI	RI%	NI	NI%	NF	NF%
20 minutes whole blood clotting	89	75.4	21	17.8	0	0.0	8	6.8	0	0.0
Complete blood count	83	70.3	26	22.0	2	1.7	7	5.9	0	0.0
Bleeding time /clotting time	97	82.2	15	12.7	0	0.0	5	4.2	1	0.8
Blood urea / creatinine & electrolyte, RFT and LFT	78	66.1	27	22.9	2	1.7	11	9.3	0	0.0
Blood grouping & Rh typing	30	25.4	59	50.0	9	7.6	20	16.9	0	0.0
Immuno-diagnosis	3	2.5	18	15.3	9	7.6	52	44.1	36	30.5
ECG	17	14.4	64	54.2	15	12.7	22	18.6		0.0
Serum CPK	28	23.7	46	39.0	6	5.1	30	25.4	8	6.8
Urine R/E	43	36.4	49	41.5	7	5.9	19	16.1	0	0.0

M = Mentioned, AP = Accepted when provoked, RI = Rejected the idea, NI = No idea, NF = No facility and respective abbreviation with % indicates percentage of abbreviated response.

### Complication after injecting antivenom

Ninety percent (*n* = 106) of respondent mentioned allergies or edema and 65% (*n* = 77) said early anaphylaxis (urticarial, dyspnea and hypotension) are complication after injecting ASV (Table 10).

**Table 10:** Probable complication after injecting anti-venom.

Knowledge about complication of injecting anti-venom	M	M%	AP	AP%	RI	RI%	NI	NI%
Early anaphylaxis (urticarial, dyspnea and hypotension)	77	65.3	21	17.8	1	0.8	19	16.1
Diarrhea and vomiting	20	16.9	53	44.9	10	8.5	35	29.7
Pyrogenic reaction (fever & chill)	21	17.8	64	54.2	3	2.5	30	25.4
Allergies / edema	106	89.8	2	1.7	0	0.0	10	8.5

M = Mentioned, AP = Accepted when provoked, RI = Rejected the idea, NI = No idea and respective abbreviation with % indicates percentage of abbreviated response.

Eighty percent (*n* = 94) of responding health workers said infection in snakebite wound is caused by cutting of wound (Table 11). After administering ASVS, 56% (*n* = 66) of health workers said blood pressure of patient should be principally monitored. Also 66% (*n* = 78) of health worker said tissue necrosis is not caused by kidney failure, cardiac arrest and hypertension.

**Table 11:** Complications of after snakebite.

Question	Answers	Count	%
Infection in snakebite wound caused by	Immobilization of bitten limb	17	14.4
	False assurance to patient to calm him	2	1.7
	Cleaning the wound with clean water	4	3.4
	Cutting of wound	94^a^	79.7
	No idea	1	0.8
After administering ASVS, patient should be principally monitored for	Bleeding	27	22.9
	Pain	0	0.0
	Edema / Dropsy	18	15.3
	Blood pressure	66^a^	55.9
	No idea	7	5.9
Tissue necrosis is resultant of snakebite caused	Kidney failure	15	12.7
	Cardiac arrest	18	15.3
	Hypertension	0	0.0
	None of the above	78^a^	66.1
	No idea	7	5.9

^a^ indicates the correct option, % = percentage among total.

### Relation of Dzongkhag wise health workers and their level of knowledge

There was association between Dzongkhag wise health workers and their level of knowledge (*r* = 0.369, *p*<0.05) (S2 Table). There was a significant difference between the knowledge score of health workers on snakebite management among the 10 Dzongkhags [*F*_(9,108)_ = 2.975, *p<*0.05] (Table 12). Health workers from Central Regional Referral Hospital (CRRH) in Sarpang had the highest (*m* = 78, *SD* = 11.28) average score of knowledge while Pemagatshel had the lowest (*m* = 55, *SD* = 11.04) (S3 Table). These results indicate that the level of knowledge among health workers from different hospitals based on Dzongkhags are not at the same level.

**Table 12:** ANOVA for knowledge scores among Dzongkhags’ health workers.

	*ss*	*df*	*ms*	*F*
Between Groups	4,776.563	9	530.729	2.975*
Within Groups	19264.767	108	178.377	
Total	24,041.331	117		

*significant at *p*<0.01, *ss* = Sum of squares, *df* = Degree of freedom, *ms* = Mean square, *F* = F-ratio.

### Relation of occupation with level of knowledge

There was significant negative association between profession and knowledge score (*r* = -0.386, *p*<0.01). When adequate and inadequate knowledge were cross-tabulated with professions, the association was significant (*r* = 34.069, *p*<0.001) (S2 Table). Among 32 Doctors, 63% (*n* = 20) were found to have adequate knowledge. Among 77 Nurses, only 10% (*n* = 8) of them had adequate knowledge. There was a significant difference between the knowledge score of health workers on snakebite management for the three professional groups [*F*_(2,115)_ = 16.284, *p*<0.001] (S4 Table). Doctors had higher mean score on knowledge {74.28 (*SD* = 11.203)}. The difference could be related to their frequent use of knowledge in treating the patients and their constant need to update knowledge using internet.

### Relation of source of expertise with level of knowledge

**Fig 2:**
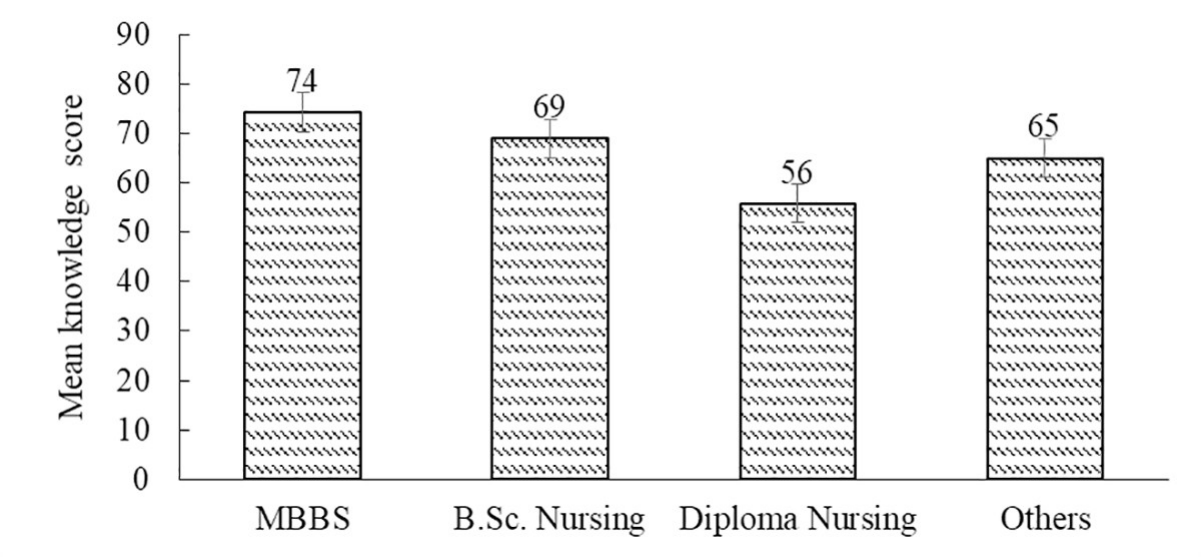
Mean of score by the source of expertise with an error bar.

There was significant association between the knowledge score and sources of expertise or qualification {MBBS, B.Sc. Nursing, Diploma Nursing and others (EMT, HA and Lab technician)}; *r* = -0.446, *p*<0.001 (S2 Table). There was a significant effect of the training courses attended and sources of the health workers’ knowledge on knowledge score [*F*_(3,114)_ = 17.876, *p<*0.001] (S5 Table). MBBS had the highest score (*m* = 74, *SD* = 11) followed by B.Sc. Nursing (*m* = 69, *SD* = 12) (Fig 2), and others *(m =* 65, *SD* = 16). Respondents who were trained under diploma nursing course scored the lowest *(m =* 56, *SD* = 11).

### Relation of gender with level of knowledge

There was significant association between gender and knowledge score (*r* = -0.394, *p<*0.001). When the levels of knowledge were cross-tabulated with gender, the association was significant (*r* = 7.12, *p*<0.05) (S2 Table). Among 73 male and 45 female respondents, 33% (*n* = 24/73) males had adequate knowledge compared to only 11% (*n* = 5/45) females. All female respondents having adequate knowledge are Doctors and none of the female nurses had adequate knowledge. Sixty five percent (*n* = 15/32) of male Doctors and 24% (8/33) of male nurses had adequate knowledge. The level of knowledge was lower in female strata of health workers compared to that of male.

### Relation of age and experience with level of knowledge

Health workers with experience of more than 10 years had higher (*m* = 65.77, *SD* = 14.49) knowledge score while those having less experience had lower (*m* = 61.71, *SD* = 14.09) (S6 Table). Age group below 35 years had lower knowledge score (*m* = 62.97, *SD* = 13.86), while those above 35 years scored more (*m* = 64.70, *SD* = 16.03) (S7 Table). This could be due to increased exposure and experience with snakebite cases with increasing age. However, there was no association between age and knowledge score (*r* = .094, *p* = 0.310). Similarly, experience of health workers showed no association with knowledge score (*r* = -.015, *p* = 0.87).

### Relation of number of snakebite cases managed with knowledge score

Average number of snakebites managed by respondents was 9 (*SD* = 13.16) and average of knowledge score was 63% (*SD* = 14.34) (S8 Table). Health workers managing more than five snakebite cases had better knowledge score (*m* = 66.27, *SD* = 13.97) than that of those who managed less than five cases and those who did not manage any. There was significant correlation between the number of snakebites managed and knowledge score (*r* = 0.223, *p* = 0.01).

### Treatment seeking behaviour

From 118 participants, 36% (*n* = 42) observed snakebite victims with cut in the bite sites made for suction. While 29% (*n* = 34) of the health workers found victims whose snakebite wound were actually sucked out. Seventy five (64%) health workers asserted that snakebite victims visited local or traditional healer before they reached hospital. A total of 60 (51%) health workers observed patients using treatment by local healers and black stone called *Jhhar Mauro* (stone believed to absorb all the snake venom and detach itself from wound once the venom is emptied from the snakebite victim). Application of green herbal paste made up of *Khenpa Shing* (*Artemisia myriantha*) and consumption of fluid from Neem leaf (*Azadirachta indica*) were also observed.

Majority (80%, *n* = 95) of the health workers encountered patients who tied bitten limbs using ropes, elastics and clothes as tourniquet to stop venom flow in other parts of body due to wrong belief. Health workers encountered patients using pressure bandage which constituted eight percent (*n* = 10). Only five percent (*n* = 6) of the health workers observed patients using WHO recommended first aid—‘Immobilization of limb’ with bandage and splint. Health workers in BHUs or primary health units might have applied these first aid measures before referral to Dzongkhag, General and Referral Hospital.

**Fig 3:**
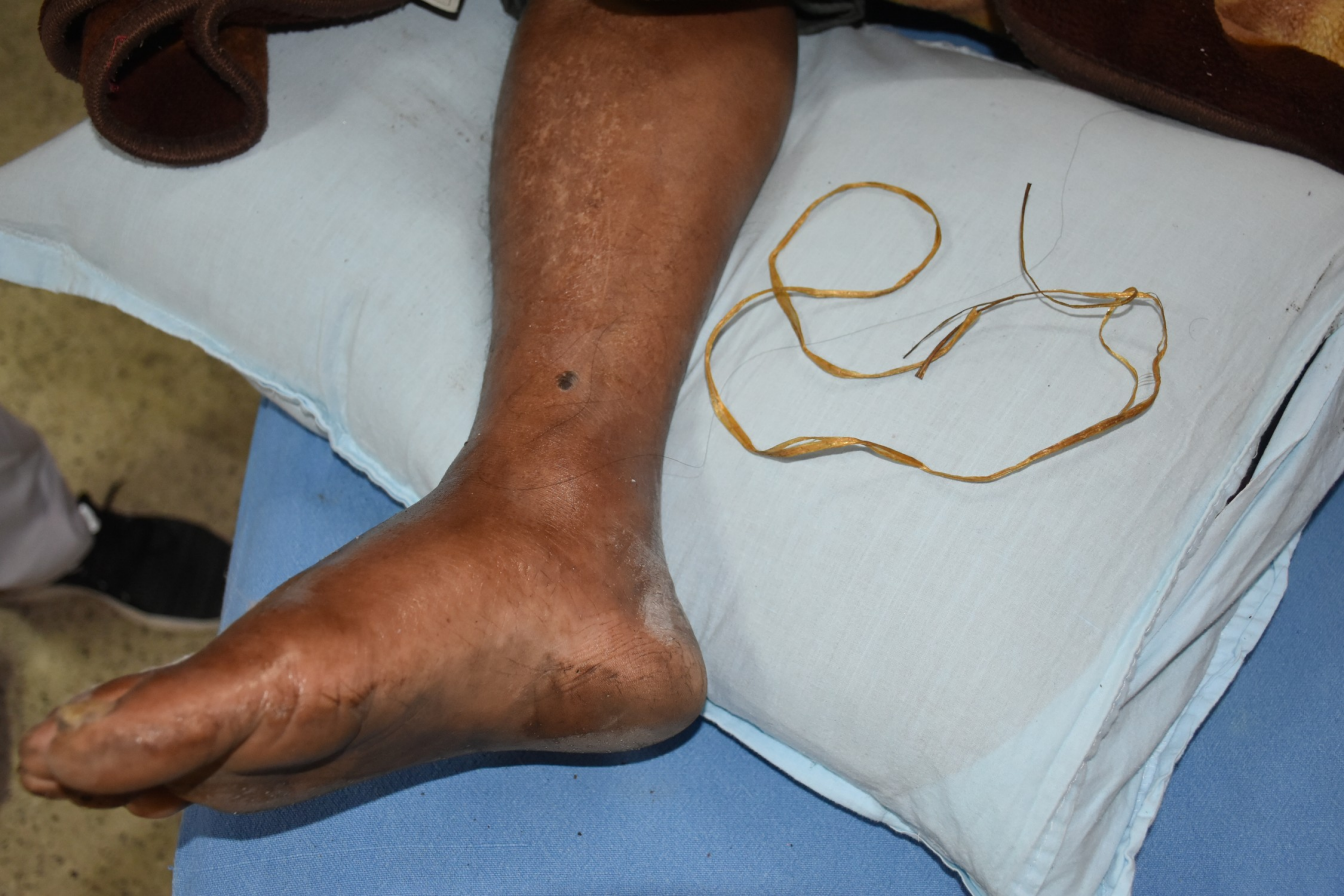
Plastic rope used as a tight tourniquet by a patient brought to Phuntsholing General Hospital.

Health workers observed in most cases, snakebite victims used tight tourniquet as a first aid measure for snakebite management. In Phuntsholing General Hospital, application of honey over the snake bitten limb along with use of tourniquet was documented during the study (Fig 3).

**Fig 4:**
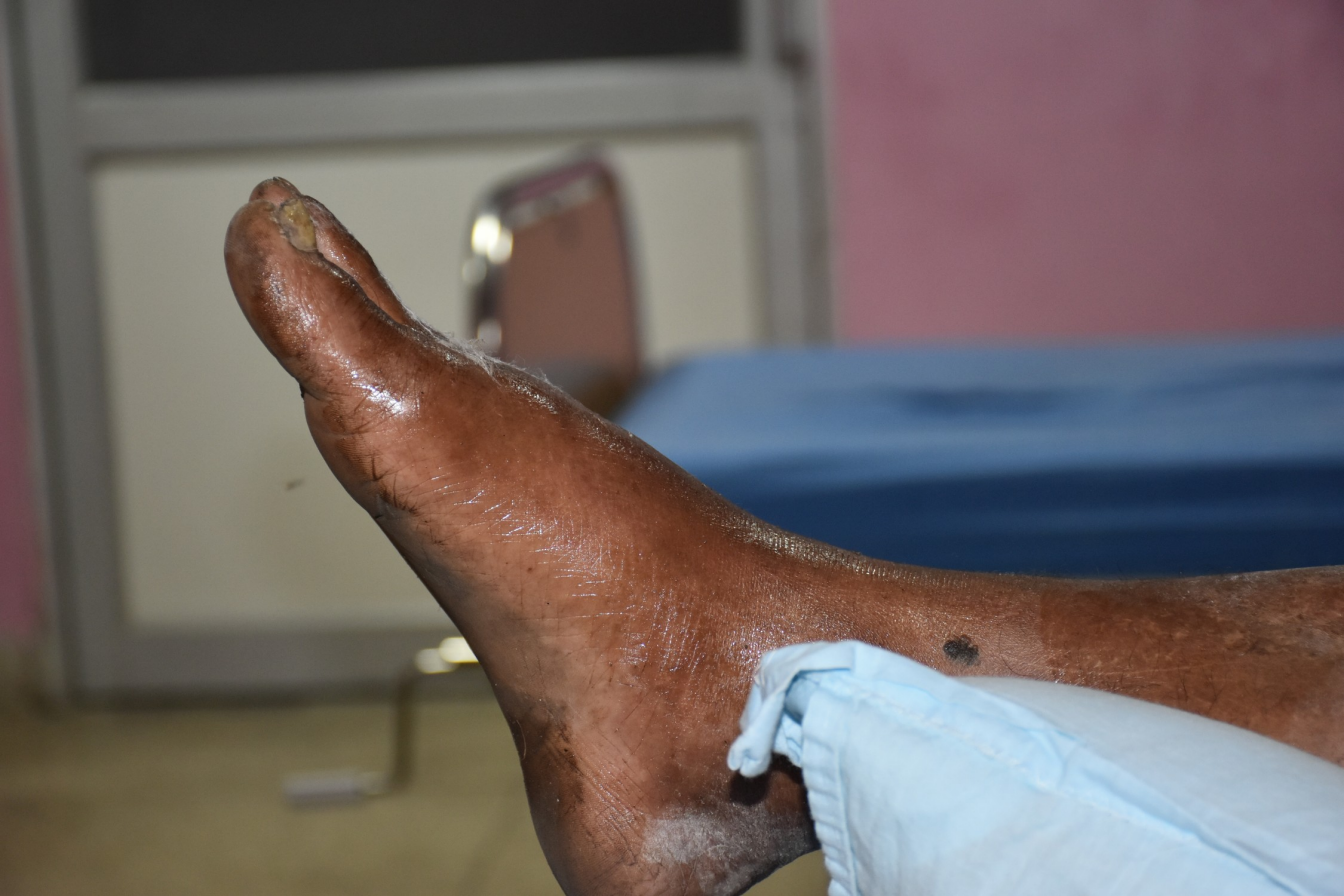
Shiny fluid is honey applied by a patient brought to Phuntsholing General Hospital.

The patient was bitten by snake at 12:00 noon but reached the hospital at 18:00 pm in the evening with swollen limb. The attendee explained that the snake was green and it may not cause much harm as this type of bites are treated with locally available medication such as honey and *Khenpa Shing* leaf paste (Fig 4).

## Discussion

Snake-human conflicts cause hundreds of snakebites which are emergency medical conditions in Bhutan. However, the study has revealed that the health workers were not well prepared for the management of snakebite. Since snakebite causes significant morbidity and mortality in Bhutan [12], health workers should be empowered with adequate knowledge and community need to be educated not to depend on traditional healing of snakebite. Further, WHO recommended measures for prevention and control of snakebite should be advocated to the health workers in Bhutan [14,20–23].

This study also revealed that health workers in Bhutan lack snake identification skills and have no idea about key identification features of kraits and vipers. Respondents identified photographs depicting big or colorful snakes as venomous (Table 3).

In addition to their empowerment, there is a need to explore relation of socio-demographic variables of health workers with their level of knowledge on snakebite management more extensively to contribute to snakebite management in Bhutan.

The study also has revealed that conservation threats exist in Bhutan because snakes are killed after snakebites if they are found. Among 118 health workers, 64% (*n* = 76) observed snakebite patients or their attendants presenting the head-smashed-dead snake or dead snakes’ photographs with smashed head in hospital for identification.

### The level of knowledge

The proportion of health workers with adequate knowledge on snakebite management in Bhutan is 25% (*n* = 29) (S2 Table), which is lower than that reported from Lao PDR [24], North Cape and Hong Kong [25,26]. A study from Bangladesh had concluded that the health workers lacked required knowledge, skill, and experience to treat snakebite victims [19]. The opportunity of being exposed to snakebite cases influenced the level of knowledge and skills required to manage snakebite in Bhutan. The number of reported snakebite cases varied from 23% (*n* = 87/391 in Sarpang to 15% (*n* = 59/391) in between 2013–2018 [3].

Similar to our findings of Doctors with higher level of knowledge compared to other health staffs, a similar study in Lao PDR also reported better knowledge of physicians over Nurses in managing snakebites[24]. Among Doctors, 78% (*n* = 25/118) responded that they used internet, social media, radio and television to search information and learn about snakes and snakebite management. Easy access to internet helps doctors to be updated with information on knowledge and skills newly suggested by WHO Guidelines and other relevant sources on snakebite management. However, due to less knowledge and skill to manage snakebite cases and snake identification, there is an immediate requirement of training for nurses to reduce the gap. Inclusion of effective training materials on snake identification and snakebite management compatible for the in-country training curricula of health workers could prove useful in narrowing the knowledge gap.

Current textbooks and medical education materials lack adequate focus in area of ASV use, complication after administration of ASV and methods to deal regional snakebites [27]. The study also revealed prevalence of incorrect information on the snakebite first aid measures incorporated in the school and university teaching materials in Nepal [28]. Therefore, precaution should be taken to include correct knowledge and skills in the training curricula.

Health workers receiving training achieved significantly better snake identification results compared to those without training opportunity. Therefore, inclusion of snakebite courses in curriculum of University and Health Science College may be useful to guarantee imparting appropriate knowledge and skills for medical graduates [24]. A study done among Nigerian health workers reported that gender does not have relation with levels of knowledge, but reported relation between knowledge score on snakebite management with the number of snakebites managed [29].

### Health seeking behavior

Consumption of *Artemisia* extract in snakebite treatment is unique and was sighted in a case at Phuntsholing General Hospital. The attendant of the snakebite victim mentioned that “we let the victim drink fluid made up of *Artemisia* and applied honey of bee called “Puttka” that absorbs venom out of the bitten limb. Application of honey in snakebite may be another long believed malpractice prevalent in rural areas. Honey has anti-bacterial properties that could prevent bacterial infection though. But both the application of honey and consumption of extract made up of *Artemisia* and Neem needs scientific scrutiny.

In Sri Lanka, 43.3% (*n* = 301) snakebite victims went for local or traditional treatment [30]. Similarly, snakebite victims in Nepal consulted traditional healers before reaching hospital due to lack of transport and difficult access to hospital [31–36]. Likewise, 80% of the snakebite victims used traditional medicine prior to admission in a hospital in South Africa [37].

Use of herbal and non-herbal medication is reported from Sri Lanka, Kenya and south-east Asia [38–40]. Similarly, use of black stone or serpent stone is believed to improve the medical efficacy from ancient time, which is believed to have come from ancient Indian tradition, the use of which is now reported from almost all parts of the snakebite prone regions of the world [41]. Observation of using such practices of traditional treatments over allopathic medicine is similar to the behaviour of Bhutanese snakebite victims observed by the health workers in Bhutan. Use of such traditional practices may be due to the need to negotiate difficult terrain and lack of quick transport facilities in reaching the nearest hospital in time. Also, during the rainy season, when snakebites are frequent, the village farm roads, which are not black topped, are either damaged by landslides or washed by floods making the accessibility difficult.

Use of traditional treatments and wrong first aid in case of snake envenomation in low and middle income countries are time consuming which convert snakebite morbidity to mortality [42,43]. An analysis of snakebite victims from Andhra Pradesh, India concluded that availability of better facilities at primary health centres with rapid transportation facilities and early administration of the polyvalent anti-venom reduce morbidity and mortality [44].

Hospital- and community-based nationally representative studies are essential to support to design antivenom against Bhutanese snakebite. Further, snake species involved in human envenomations in Bhutan needs additional systematic studies [1,3]. Snakebite victims die trying to reach referred hospitals and in hospitals at bordering town in India [45]. A case study published from Bhutan, “Is anti-snake venom required for all snakebites?”, explains confusion due to lack of national snakebite management guidelines [46]. Also, in the Indian subcontinent including Bhutan, snakebite victims use medicinal plants for treatment of snakebites which requires scientific validation [47,48].

Patients with nonvenomous snakebites stay many days in hospital due to use of tight tourniquet which induces ischemia causing gangrene [3]. Use of wrong first aid results in morbidity/mortality of victims and wastes health workers' time and effort. Creating awareness among general people, preparation of training materials based on knowledge of local snakes known, and providing appropriate trainings to all levels of health workers will obviously improve snakebite management cases in the country.

### Limitations of the study

We were ignorant about the level of knowledge of care givers in remote areas of Bhutan. Knowledge of medical workers included in this study may not be wholly representative nationwide because of use of non-random sampling method. Purposive sampling of the respondents might introduce some unseen biases.

## Conclusion

The level of knowledge of health workers in Bhutan on snake identification and snakebite management was inadequate. Since physicians exposed to the snakebite cases frequently had better knowledge and skills in managing snakebite cases, the trained/empowered health persons should be employed for long term in health institutions in snakebite prone regions. Prevalent use of traditional medication practices and less use of WHO recommended first aid measures suggest educational opportunities to overcome obstacles for snakebite management in Bhutan. A multi-center, hospital-based prospective study of snakebites in Bhutan is essential to document mortality and morbidity due to snakebites more comprehensively.

## Supporting information

S1 File Structured Questionnaire used in survey. (PDF)

S2 File Informed Consent Form. (PDF)

S1 Table Socio-demographic features of respondents. (DOCX)

S2 Table Cross tabulation of socio-demographic variables with level of knowledge. (DOCX)

S3 Table Difference in mean score among Dzongkhags. (DOCX)

S4 Table ANOVA of knowledge among Doctors, Nurses and Others. (DOCX)

S5 Table ANOVA of knowledge score among different source of expertise. (DOCX)

S6 Table Mean Score by years of experience. (DOCX)

S7 Table Mean score by age. (DOCX)

S8 Table Mean Score per averaged number of snakebite managed by health workers. (DOCX)

S1 Fig *Boiga multifasciata* (Photo by Sunil Sapkota). (JPG)

S2 Fig *Ptyas mucosa* (Photo by Sunil Sapkota). (JPG)

S3 Fig *Bungarus fasciatus* (Photo provided by Vivek Sharma). (JPG)

S4 Fig *Daboia russelii* (Photo provided by Vivek Sharma). (JPG)

S5 Fig *Trimeresurus albolabris* (Photo Provided by Narayan Sapkota). (JPG)

S6 Fig *Amphiesma stolatum* (Photo by Sunil Sapkota). (JPG)

S7 Fig *Amphiesma platyceps* (Photo by Sunil Sapkota). (JPG)

S8 Fig *Python molurus bivittatus* (Photo by Sunil Sapkota). (JPG)

S9 Fig *Bangarus niger* (Photo by Sunil Sapkota). (JPG)

S10 Fig *Ptyas nigromarginata* (Photo by Sunil Sapkota). (JPG)

S11 Fig *Naja kaouthia* (Photo by Sunil Sapkota). (JPG)

S12 Fig *Xenochrophis piscator* (Photo by Sunil Sapkota). (JPG)

S13 Fig *Oligodon juglandifer* (Photo by Sunil Sapkota). (JPG)

S14 Fig *Rhabdophis subminiatus* (Photo by Narayan Sapkota). (JPG)

S15 Fig *Pseudoxenodon macrops* (Photo by Sunil Sapkota). (JPG)

S16 Fig *Ovophis monticola* (Photo by Dhan B. Gurung). (JPG)

S17 Fig *Naja naja* (Photo provided by Vivek Sharma). (JPG)

S18 Fig *Lycodon aulicus* (Photo by Sunil Sapkota). (JPG)

S19 Fig *Ophiophagus Hannah* (Photo by Sunil Sapkota). (JPG)

S20 Fig *Bangarus lividus* (Photo downloaded from raptilefacts.com, no author information provided). (JPG)
