# Supplementary material for: Knowledge of health workers on snakes and snakebite management and treatment seeking behavior of snakebite victims in Bhutan
Source: PLoS Negl Trop Dis. 2020 Nov 30;14(11):e0008793. doi: 10.1371/journal.pntd.0008793 (PMC7728388; doi:10.1371/journal.pntd.0008793)
Supplement: S1 File — (PDF) [file pntd.0008793.s001.pdf]

Knowledge of Health Workers

Section I: Demographic features of health workers

1. Profession: Doctor / Nurse/ Pharmacy/Lab Technician/ EMT/ Medical assistance
2. Age: …..
3. Sex : Male/ Female
4. Religion: Buddhist/ Hindu/Others
5. Marital Status: Married/ Unmarried/ Divorced
6. Type of family
   i) Nuclear family
   ii) Joint family
   iii) Extended family
   iv) Single
7. Total monthly income of the family (In Nu.)
   i. 0-10,000
   ii. 10,001-30,000
   iii. 30,001- 50,000
   iv. 50,001- 1,00,000
   v. 1,00,000-5,00,000
   vi. 5,00,000-10,00,000
8. Where did you spent most of your childhood years?
   i. Rural
   ii. Urban
9. Professional experience (Years) (……………….)
10. Number of years served in rural areas (……………..)
11. Number of years served in urban areas (……………)
12. Number of snakebite managed clinically in person (…)
13. Total number of medical staffs in hospital (……..)
14. Total medical staff involved in treatment of snake bite: (………….)
15. Area of work
   i) Emergency ward
   ii) IPD
   iii) ICU
   iv) General Ward
   v) OPD
   vi) Other
16. Duration of stay in this place of residence ……………
17. Source of information about snakes and snakebite
   i) Curricular books
   ii) Tales and talks of parents, folklores and stories in my village
   iii) Internet, social media, Television and Radio Programs
   iv) Training

18. Expertise on topics related with snakes and snakebite management is gained from

i. MBBS
ii. Emergency Medical Technician
iii. Nursing
iv. B.Sc. Nursing
v. Lab Technician
vi. Health assistant course
vii. Other (…………………..)
viii. Trainings (Hours……..)

19. In your opinion, when do venomous snakebites generally occur?

i. Throughout the day
ii. During dawn and dusk
iii. Night
iv. Afternoon

20. Snakebite victim report to hospital with

i. Cuts in the bitten site
ii. Tourniquets
iii. Herbal medication applications
iv. Suction
v. Tied with rope/elastic/clothes
vi. Local healers traditional treatments
vii. Pressure bandages
viii. Immobilization with splints

21. Do people bring snake biting them in the hospital? Yes/ No (……………………..)

22. Do snakebite victims visit local healers before they reach hospital? Yes/ No (……)

23. **Rate yourself**

S.n.	Your Expertise on	Rank your Expertise				
**1.**	**Snake identification is**	Very Confident	Moderately Confident	Lack confidence	No confidence	No idea
**2.**	**Preventive measures of snake bite is**	Very confident	Moderately Confident	Lack confidence	No confidence	No idea
**3.**	**First Aid of snakebite management is**	Very confident	Moderately Confident	Lack confidence	No confidence	No idea
**4.**	**Sign and symptoms of snakebite**	Very confident	Moderately Confident	Lack confidence	No confidence	No idea
**5.**	**Lab test of snakebite management is**	Very confident	Moderately Confident	Lack confidence	No confidence	No idea
**6.**	**Complication after injecting Anti Snakevenom is**	Very confident	Moderately Confident	Lack confidence	No confidence	No idea
**7.**	**Requirement of snakebite management & snake identification training in your centre is**	Very Important	Moderately Important	Important	Not Required	No idea

24. For snake identification printed pictures will be shown

SI.	Common name	V	NV	SI.	Common name	V	NV
1				11			
2				12			
3				13			
4				14			
5				15			
6				16			
7				17			
8				18			
9				19			
10				20			

25. What is the best method to prevent snakebite around houses?

S.n.	Preventive measures of snakebite	Mentioned	Accepted when Provoked	Rejected the idea	No idea
1.	Spraying phenol				
2.	Hunting and killing snakes				
3.	Cleaning and clearing bushes, and debris laying on the ground				
4.	Spraying kerosene				
5.	Use of protective equipment				
6.	Cover up holes in surrounding				
7.	Avoid marshy and busy area				
8.	Spraying Alcohol				
9.	Spraying garlic syrup				
10.	Praying to Local Deities/god				

26. What are the Signs and symptoms of snakebite?

S.n.	Signs and symptoms of snakebite	Mentioned	Accepted when Provoked	Rejected the idea	No idea
1.	Swelling wound pain and Blisters				
2.	Dizziness and vomiting				
3.	Blurring of vision				
4.	Convulsion				
5.	Unconsciousness/mental confusion				
6.	Dropping of eyelids/Ptosis				
7.	Weakness of neck muscle				
8.	Difficulty in swallowing				
9.	Nasal regurgitation/voice				
10.	Difficulty in respiration				
11.	Bleeding from gum and vomiting				
12.	Persistent bleeding from bite site				
13.	Severe muscle pain				
14.	Dark coloured urine				
15.	Scanty or no urine output				
16.	Renal Failure				
17.	Shock/collapse				
18.	Fang Marks				

19.	Low Blood Pressure/ High Pulse Rate				
20.	Haematuria/Bruises				

27. What are the lab test for snakebite management?

S.n.	Knowledge on lab test	Mentioned	Accepted when Provoked	Rejected the idea	No idea
1.	20 minutes whole blood clotting				
2.	Complete blood count				
3.	Bleeding time Clotting time				
4.	Blood urea/ creatinine & electrolyte, RFT				
5.	Blood grouping & Rh typing				
6.	Immuno-diagnosis				
7.	ECG				
8.	Serum CPK				
9.	Urine R/E				
10.	LFT				

28. What are the probable complication after injecting anti-venom?

S.n.	Knowledge about complication of injecting anti-venom	Mentioned	Accepted when Provoked	Rejected the idea	No idea
1.	Early anaphylaxis (urticarial, dyspnea and hypotension)				
2.	Diarrhoea and vomiting				
3.	Pyrogenic reaction(fever &chill)				
4.	Allergies/ Oedema				

(Select the best answer.)

**Section II: Snake Identification**		
29.	Which of the following key feature of snake help you to identify Common Cobra?	i. Shiny color<br>ii. Paired white bands along the body<br>iii. Black head with brown body<br>iv. Spectacle present behind the hood<br>v. No idea
30.	Which of the following key feature of snake help you to identify kraits?	i. Paired white bands along the body<br>ii. Large hexagonal mid-dorsal cells<br>iii. Black head with brown body<br>iv. Spectacle present behind the hood<br>v. No idea
31.	Which of the following key feature of snake help you to identify Vipers?	i. Triangular head with irregular scales<br>ii. Large hexagonal mid-dorsal cells<br>iii. Black head with brown body<br>iv. Spectacle present behind the hood<br>v. No idea
**Section III : First Aid for snakebite**		
32.	Appropriate site for tourniquet is	i. Over the site of bite<br>ii. 2 inches above the site of bite<br>iii. 2 inches below the site of bite<br>iv. Should not be used<br>v. No idea
33.	Bite mark should be covered with bandage	i. Yes<br>ii. No<br>iii. No idea
34.	When a snakebite victim reports to hospital you will	i. Apply of pressure bandage<br>ii. Slice the wound<br>iii. Suck the wound<br>iv. Reassure and calm the patient<br>v. No idea
35.	Reassuring and calming of the snakebite victim helps to	i. Prevents complication and helps in observing sign and symptom<br>ii. Reduces bleeding<br>iii. Reduces rate of venom diffusion<br>iv. Induces sleep<br>v. No idea
36.	Best suggestion to manage future snakebite properly is	i. Find local healers<br>ii. Take victim in quick and comfortable transport to nearest hospital provided with ASVS<br>iii. Find the snake and kill it for identification<br>iv. Go to the nearest clinic<br>v. No idea

Section IV: Signs and symptoms of snakebite		
37.	Cobra venom mainly causes	i. Nephrotoxic effect<br>ii. Neurotoxic effect<br>iii. Cytotoxic effect<br>iv. Hemotoxic effect<br>v. No idea
38.	Venomous and poisonous are similar	i. Yes<br>ii. No<br>iii. No idea
39.	Viper snakebite mainly causes	i. Nephrotoxic effect<br>ii. Neurotoxic effect<br>iii. Cytotoxic effect<br>iv. Hemotoxic effect<br>v. No idea
40.	How can you declare non-venomous snakebites if any snakebite victim arrives in your centre?	i. Swelling, pain on bitten site<br>ii. Wound with many dots<br>iii. Drooping eyes with scratch/two dots and broken neck symptoms<br>iv. After the observation if no symptoms occur<br>v. No idea
**Section VII: Complication of snakebite**		
41.	Infection in snakebite wound caused by	i. Immobilization of bitten limb<br>ii. False assurance to patient to calm him<br>iii. Cleaning the wound with clean water<br>iv. Cutting of wound<br>v. No idea
42.	Tissue necrosis is resultant of snakebite caused	i. Kidney failure<br>ii. Cardiac arrest<br>iii. Hypertension<br>iv. None of the above<br>v. No idea
43.	Tissue death at bitten site is generally caused by	i. Cobra bite<br>ii. Wolf snake bite<br>iii. Rat Snake bite<br>iv. Krait bite<br>v. No idea
44.	Respiratory failure is mostly caused by	i. Krait bite<br>ii. Rat snake bite<br>iii. Python bite<br>iv. Wolf snake bite<br>v. No idea
45.	Important symptoms of neurotoxic envenomation is	i. Vomiting<br>ii. Nausea<br>iii. Drooping of eyelids<br>iv. Body pain<br>v. No idea

46.	Polyvalent ASVS can be administered for	i. When patients develops symptoms of envenomation
		ii. Snakebite patient has fang marks
		iii. required after all snakebite
		iv. Snake bite patients is in fear and unconsciousness
		v. No idea
47.	After administering ASVS, patient should be principally monitored for	i. Bleeding
		ii. Pain
		iii. Oedema/Dropsy
		iv. Blood pressure
		v. No idea

Important Notes

48. Stories/myths/legends (snakes and snakebite management)……………………………
