# Supplementary material for: Knowledge of health workers on snakes and snakebite management and treatment seeking behavior of snakebite victims in Bhutan
Source: PLoS Negl Trop Dis. 2020 Nov 30;14(11):e0008793. doi: 10.1371/journal.pntd.0008793 (PMC7728388; doi:10.1371/journal.pntd.0008793)
Supplement: S2 File — (PDF) [file pntd.0008793.s002.pdf]

Appendix III
Consent Form Version No.: 2

Informed Consent Form for Qualitative Studies

Target Population: Health Practitioners and Snake Bite Victims (Literate member of house hold/Neighbour/Friend)

Introduction

This informed consent form is for health practitioners in Dzongkhag hospital and snakebite victims taking medication from Chhukha, Pemagatshel, Samdrupjongkhar, Samtse and Sarpang hospital who we are inviting to participate in research (Dissertation Thesis) titled "Retrospective study of snakebite, health seeking behavior of snakebite victims and knowledge of health practitioners handling snakebite cases in Bhutan"

Name of Principle Investigator: Sunil Sapkota

Name of Organization: College of Natural Resources, Lobesa, Punakha. Royal University of Bhutan

Name of Sponsor: UNESCO SAF Madanjeet Singh Foundation

Name of Project and Version: 11-27-2018-01

This Informed Consent Form has two parts:

- **Information Sheet (to share information about the study with you)**
- **Certificate of Consent (for signatures if you choose to participate)**

You will be given a copy of the full Informed Consent Form

Part I: Information Sheet

Introduction

I am Sunil Sapkota, a student of BSc Forestry final year in the College of Natural Resources, Royal University of Bhutan. I am doing research on Snakebite which is very common and is one of the life taking causes in Bhutan and in the Southeast Asia. I am going to give you information and invite you to be part of this research. You do not have to decide today whether or not you will participate in the research. Before you decide, you can talk to anyone you feel comfortable with about the research.

This consent form may contain words that you do not understand. Please ask me to stop as we go through the information and I will take time to explain. If you have questions later, you can ask them to me.

Purpose of the Research

Snakebites are killing several farming people in Bhutan annually and snakebite is a real fear/health problem to farming communities and the people depending on forest produces. Deaths due to snakebites are dependent on first aid provisions available with the health service centres and health seeking behaviour of snakebite victims. This study attempts to understand the possible causes of deaths related to snakebite cases in Bhutan, level of awareness and knowledge of snakes, and snakebite management. We believe that you can help us to save more people in future resulting from snakebites by telling us what you know about snakes, snakebite management and about local health practices in general. We want to learn about the different ways that people try to manage snakebite cases – both from the perspective of service providers as well as service seekers. In the process of data collection, we also want to understand the knowledge and skill gaps of practitioners and share information on various types of snakes which will help to improve snakebite management in Bhutan in future.

Type of Research Intervention

This research will involve your participation in a half hour interview.

Participant Selection

You are being invited to take part in this research because we feel that your experience as a health practitioners / snakebite victim can contribute much to our understanding and knowledge of local health practices, which will ultimately help in improving the snakebite management system in Bhutan.

Voluntary Participation

Your participation in this research is entirely voluntary. It is your choice whether to participate or not. If you choose not to participate all the services you receive at this Centre will continue and nothing will change.

OR

The choice that you make will have no bearing on your job or on any work-related evaluations or reports. You may change your mind later and stop participating even if you agreed earlier.

Procedures

We are asking you to help us learn more about snakebite in your community. We are inviting you to take part in this research project. If you accept, you will be asked to tell us about your knowledge on snakes and snakebite management.

For Health Practitioners

I will be asking some of your demographic data in the first section of the questionnaire and in section I will be asking question about snake identification and snake bite management practices you are adopting in your job station.

Fill out a survey which will be provided by Sunil Sapkota and collected by Sunil Sapkota. OR You may answer the questionnaire yourself, or it can be read to you and you can say out loud the answer you want me to write down.

If you do not wish to answer any of the questions included in the survey, you may skip them and move on to the next question. I will sit down with you in a comfortable place at your job station or anywhere when you are fee enough to answer my question. If it is better for you, the interview can take place in your home or a friend's home. The information recorded is confidential, your name is not being included on the forms, only a number will identify you, and no one else except Sunil Sapkota will have access to your survey.

For Snakebite Victim

I will be asking some of your demographic data in the first section of the questionnaire which will be anonymous and in second section I will be asking question about snake identification and snake bite management practices you are adopting in your community. I will be requesting you about your condition and situation you went through after snakebite and consequences developed because of snakebite.

Participate in an interview with Sunil Sapkota or myself.

During the interview, I (with translator in required situation) will sit down with you in a comfortable place at the Centre. If it is better for you, the interview can take place in your home or a friend's home. If you do not wish to answer any of the questions during the interview, you may say so and the interviewer will move on to the next question. No one else but the interviewer will be present unless you would like someone else to be there. The information recorded is confidential, and no one else except Sunil Sapkota or myself will access to the information documented during your interview. The entire interview will be tape-recorded, but no-one will be identified by name on the tape. The tape will be kept [explain how the tape will be stored]. The information recorded is confidential, and no one else except Sunil Sapkota will have access to the tapes. The tapes will be destroyed after 2 years.

Duration

The interview takes place for 30 minutes only.

Risks

There is a risk that you may share some personal or confidential information by chance, or that you may feel uncomfortable talking about some of the topics. However, we do not wish for this to happen. You do not have to answer any question or take part in the discussion/interview/survey if you feel the question(s) are too personal or if talking about them makes you uncomfortable.

Benefits

There will be no direct benefit to you, but your participation is likely to help us find out more about how to manage snakebite cases in your community. I will answer your question about snake and snakebite management and make you aware about the 10 species of common snakes of Bhutan after the survey is completed.

Reimbursement

As this research is dissertation thesis and I am student I cannot provide any incentives or money.

Confidentiality

The research being done in the community may draw attention and if you participate you may be asked questions by other people in the community. We will not be sharing information about you to anyone outside of the research team. The information that we collect from this research project will be kept private. Any information about you will have a number on it instead of your name. Only the researchers will know what your number is and we will lock that information up with a lock and key. It will not be shared with or given to anyone except Sunil Sapkota (Researcher) and College of Natural Resources, Royal University of Bhutan.

Sharing the Results

Nothing that you tell us today will be shared with anybody outside the research team, and nothing will be attributed to you by name. The knowledge that we get from this research will be shared with you and your community from the dissertation thesis and research publication. We will publish the results so that other interested people may learn from the research.

Right to Refuse or Withdraw

You do not have to take part in this research if you do not wish to do so, and choosing to participate will not affect your job or job-related evaluations in any way. You may stop participating in the Survey/interview at any time that you wish without your job being affected. I will give you an opportunity at the end of the interview to review your remarks, and you can ask to modify or remove portions of those, if you do not agree with my notes or if I did not understand you correctly.

Who to Contact

If you have any questions, you can ask them now or later. If you wish to ask questions later, you may contact any of the following: Sunil Sapkota (Email: sunilsapkota11@gmail.com, Phone: 17332805).

This proposal has been reviewed and approved by REBH, which is a committee whose task it is to make sure that research participants are protected from harm. If you wish to find about more about the IRB, contact Mr. Mangal Singh Gurung, REBH Secretariat, MOH (Phone: 17920280/3226062).

You can ask me any question about any part of the research study, if you wish to. Do you have any question?

Part II: Certificate of Consent

I have been invited to participate in research about knowledge on snakes and snakebite management. I have read the foregoing information, or it has been read to me. I have had the opportunity to ask questions about it and any questions I have been asked have been answered to my satisfaction. I consent voluntarily to be a participant in this study.

Name of Participant __________________

Signature of Participant ___________________

Date ___________________________

Day/month/year

Statement by the researcher/person taking consent

I have accurately read out the information sheet to the potential participant, and to the best of my ability made sure that the participant understands that the following will be done:

I confirm that the participant was given an opportunity to ask questions about the study, and all the questions asked by the participant have been answered correctly and to the best of my ability. I confirm that the individual has not been coerced into giving consent, and the consent has been given freely and voluntarily.

A copy of this ICF has been provided to the participant.

Signature: -----------------------------------

Sunil Sapkota (Principle investigator)

Date ___________________________

Day/month/year
