# Supplementary material for: Knowledge of health workers on snakes and snakebite management and treatment seeking behavior of snakebite victims in Bhutan
Source: PLoS Negl Trop Dis. 2020 Nov 30;14(11):e0008793. doi: 10.1371/journal.pntd.0008793 (PMC7728388; doi:10.1371/journal.pntd.0008793)
Supplement: S1 Table — (DOCX) [file pntd.0008793.s003.docx]

**S1 Table**. Socio-demographic features of respondents

| **Socio- demographic variables** |  | ***f*** | **%** |
| --- | --- | --- | --- |
| Dzongkhag | Mongar | 11 | 9.3 |
|  | Trashigang | 9 | 7.6 |
|  | Pemagatshel | 10 | 8.5 |
|  | Samdrup Jongkhar | 10 | 8.5 |
|  | Trongsa | 7 | 5.9 |
|  | Wangdue | 7 | 5.9 |
|  | Samtse | 15 | 12.7 |
|  | Punakha | 9 | 7.6 |
|  | Chhukha | 26 | 22 |
|  | Sarpang | 14 | 11.9 |
| Profession | Doctors | 32 | 27.1 |
|  | Nurse | 77 | 65.3 |
|  | Others | 9 | 7.6 |
| Sex | Male | 73 | 61.9 |
|  | Female | 45 | 38.1 |
| Religion | Buddhism | 93 | 78.8 |
|  | Hindu | 20 | 16.9 |
|  | Others | 5 | 4.2 |
| Marital status | Married | 89 | 75.4 |
|  | Unmarried | 29 | 24.6 |
| Type of family | Nuclear | 67 | 56.8 |
|  | Joint | 16 | 13.6 |
|  | Extended | 6 | 5.1 |
|  | Single | 29 | 24.6 |
| Income in Ngultrum (Nu.) | 10,000-30,000 | 92 | 78 |
|  | 30,000-50,000 | 21 | 17.8 |
|  | 50,000-70,000 | 5 | 4.2 |
| Residence in childhood | Rural | 63 | 53.4 |
|  | Urban | 55 | 46.6 |
| Expertise source | MBBS | 32 | 27.1 |
|  | B.Sc. Nursing | 14 | 11.9 |
|  | Diploma Nursing | 59 | 50.0 |
|  | Others | 13 | 11.0 |

f=frequency, %=percentage form total
