# Supplementary material for: Knowledge of health workers on snakes and snakebite management and treatment seeking behavior of snakebite victims in Bhutan
Source: PLoS Negl Trop Dis. 2020 Nov 30;14(11):e0008793. doi: 10.1371/journal.pntd.0008793 (PMC7728388; doi:10.1371/journal.pntd.0008793)
Supplement: S2 Table — (DOCX) [file pntd.0008793.s004.docx]

**S2 Table.** Cross tabulation of socio-demographic variables with level of knowledge

|  | **Demographics** | **IK(%)** | **AK(%)** | **Total(%)** | **x2** | **cc/r** | **df** | **P** | **Remarks** |
| --- | --- | --- | --- | --- | --- | --- | --- | --- | --- |
| Dzongkhag | Mongar | 7 (63.6) | 4 (36.4) | 11 (9.3) | 18.579 | 0.369 | 9 | 0.029 | 9 cells (45.0%) have expected count less than 5. The minimum expected count is 1.72. |
|  | Trashigang | 8 (88.9) | 1 (11.1) | 9 (7.6) |  |  |  |  |  |
|  | Pemagatshel | 10 (100) | 0 (0.0) | 10 (8.5) |  |  |  |  |  |
|  | Samdrup Jongkhar | 6 (60) | 4 (40.0) | 10 (8.5) |  |  |  |  |  |
|  | Trongsa | 7(100) | 0 (0.0) | 7 (5.7) |  |  |  |  |  |
|  | Wangdue | 5 (71.5) | 2 (28.6) | 7 (5.7) |  |  |  |  |  |
|  | Samtse | 13 (86.7) | 2 (13.3) | 15 (12.7) |  |  |  |  |  |
|  | Punakha | 8 (88.9) | 1 (11.1) | 9 (7.6) |  |  |  |  |  |
|  | Chhukha | 19 (73.1) | 7 (26.9) | 26 (22) |  |  |  |  |  |
|  | Sarpang | 6 (42.9) | 8 (57.1) | 14 (11.9) |  |  |  |  |  |
| Profession | Doctor | 12 (37.5) | 20 (62.5) | 32 (27.1) | 34.608 | 0.473 | 2 | <.001 | 1 cells (16.7%) have expected count less than 5. The minimum expected count is 2.21. |
|  | Nurse | 69 (89.6) | 8(10.4) | 77 (65.3) |  |  |  |  |  |
|  | Others | 8 (88.9) | 1 (11.1) | 9 (7.6) |  |  |  |  |  |
| Sex | Male | 49 (67.1) | 24(32.9) | 73 (61.9) | 7.115 | 0.238 | 1 | <.01 | 0 cells have expected count less than 5. The minimum expected count is 11.6. |
|  | Female | 40 (88.9) | 5 (11.1) | 45 (38.1) |  |  |  |  |  |
| Religion | Buddhism | 70(75.3) | 23 (24.7) | 93 (78.8) | 0.869 | 0.085 | 2 | 0.648 | 3 cells (50 %) have expected count less than 5. The minimum expected count is 1.23. |
|  | Hinduism | 16 (80.0) | 4 (20) | 20 (16.9) |  |  |  |  |  |
|  | Others | 3 (60.0) | 2 (40) | 5 (4.2) |  |  |  |  |  |
| Marital Status | Married | 68 (76.4) | 21 (23.6) | 89 (75.4) | 0.188 | 0.040 | 1 | 0.665 | 0 cells have expected count less than 5. The minimum expected count is 7.13. |
|  | Unmarried | 21 (72.4) | 8 (27.6) | 29 (24.6) |  |  |  |  |  |
| Current family Status | Nuclear | 51 (76.1) | 16 (23.9) | 67 (56.8) | .700 | 0.077 | 3 | 0.873 | 3 cells (37.5 %) have expected count less than 5. The minimum expected count is 1.47. |
|  | Joint | 13 (81.3) | 3 (18.7) | 16 (13.6) |  |  |  |  |  |
|  | Extended | 4 (66.7) | 2 (33.3) | 6 (5.1) |  |  |  |  |  |
|  | Single | 21 (72.4) | 8 (27.6) | 29 (24.6) |  |  |  |  |  |
| Family Income in (1000 Nu.) | <30 | 72(78.3) | 20(21.7) | 92 (78) | 1.910 | 0.126 | 2 | 0.385 | 3 cells (50 %) have expected count less than 5. The minimum expected count is 1.14. |
|  | 30-50 | 14 (66.7) | 7 (33.3) | 21 (17.8) |  |  |  |  |  |
|  | >50 | 3 (60) | 2 (40.0) | 5 (4.2) |  |  |  |  |  |
| Childhood spent in | Rural | 49(77.8) | 14(22.4) | 63 (53.4) | 0.404 | 0.058 | 1 | 0.525 | 3 cells (50 %) have expected count less than 5. The minimum expected count is 13.52. |
|  | Urban | 41 (74.5) | 14 (25.5) | 55 (46.6) |  |  |  |  |  |

| Sources of knowledge | MBBS | 12 (37.5) | 20 (62.5) | 32 (27.12) | 42.446 | 0.514 | 3 | <.001 | 2 cells (25%) have expected count less than 5. The minimum expected count is 3.19. |
| --- | --- | --- | --- | --- | --- | --- | --- | --- | --- |
|  | B.Sc. Nursing | 9 (64.3) | 5 (35.7) | 14 (11.86) |  |  |  |  |  |
|  | Diploma Nursing | 58 (98.3) | 1 (1.7) | 59 (50) |  |  |  |  |  |
|  | Others | 10 (76.9) | 3 (23.1) | 13 (11.02) |  |  |  |  |  |
|  | Total | 89(75.4) | 29 (24.6) | 118 (100) |  |  |  |  |  |

IK= Inadequate knowledge and AK= Adequate Knowledge, cc=correlation coefficient, r=Pearson coefficient, df=degree of freedom, P=p-value
