# Supplementary material for: Knowledge of health workers on snakes and snakebite management and treatment seeking behavior of snakebite victims in Bhutan
Source: PLoS Negl Trop Dis. 2020 Nov 30;14(11):e0008793. doi: 10.1371/journal.pntd.0008793 (PMC7728388; doi:10.1371/journal.pntd.0008793)
Supplement: S3 Table — (DOCX) [file pntd.0008793.s005.docx]

**S3 Table:** Difference in mean score among Dzongkhags

| Dzongkhag | *n* | *m* | *SD* | *SE* |
| --- | --- | --- | --- | --- |
| Mongar | 11 | 67.09 | 13.134 | 3.960 |
| Trashigang | 9 | 59.11 | 12.494 | 4.165 |
| Pemagatshel | 10 | 55.00 | 11.045 | 3.493 |
| Samdrup Jongkhar | 10 | 66.80 | 11.858 | 3.750 |
| Trongsa | 7 | 59.71 | 7.910 | 2.990 |
| Wangdue Phodrang | 7 | 64.00 | 11.136 | 4.209 |
| Samtse | 15 | 57.13 | 13.043 | 3.368 |
| Punakha | 9 | 62.89 | 11.826 | 3.942 |
| Chhukha | 26 | 61.96 | 17.494 | 3.431 |
| Sarpang | 14 | 77.79 | 11.288 | 3.017 |

*m*= Mean Knowledge Score*, n* = number of respondents, *SD* = Standard Deviation, *SE* = Standard Error
