# Supplementary material for: Knowledge of health workers on snakes and snakebite management and treatment seeking behavior of snakebite victims in Bhutan
Source: PLoS Negl Trop Dis. 2020 Nov 30;14(11):e0008793. doi: 10.1371/journal.pntd.0008793 (PMC7728388; doi:10.1371/journal.pntd.0008793)
Supplement: S5 Table — (DOCX) [file pntd.0008793.s007.docx]

**S5 Table.** ANOVA of knowledge score among different source of expertise

|  | ***ss*** | ***df*** | ***ms*** | ***F*** |
| --- | --- | --- | --- | --- |
| Between Groups | 7691.255 | 3 | 2563.752 | 17.876^*^ |
| Within Groups | 16350.075 | 114 | 143.422 |  |
| Total | 24041.331 | 117 |  |  |

*significant at p<0.01, *ss*=Sum of squares, *df*=Degree of freedom, *ms*=Mean square, *F*=F-ratio
