# Supplementary material for: Knowledge of health workers on snakes and snakebite management and treatment seeking behavior of snakebite victims in Bhutan
Source: PLoS Negl Trop Dis. 2020 Nov 30;14(11):e0008793. doi: 10.1371/journal.pntd.0008793 (PMC7728388; doi:10.1371/journal.pntd.0008793)
Supplement: S6 Table — (DOCX) [file pntd.0008793.s008.docx]

**S6 Table.** Mean Score by years of experience

| **Years of Experience** | ***m*** | ***N*** | ***SD*** | ***SE*** |
| --- | --- | --- | --- | --- |
| Ten years or less | 61.71 | 70 | 14.091 | 1.684 |
| More than ten years | 65.77 | 48 | 14.493 | 2.092 |
| Overall average | 63.36 | 118 | 14.335 | 1.320 |

*m*= Mean Knowledge Score*, N*=Number of health workers, *SD*= Standard deviation, *SE*= Standard error
