# Supplementary material for: Knowledge of health workers on snakes and snakebite management and treatment seeking behavior of snakebite victims in Bhutan
Source: PLoS Negl Trop Dis. 2020 Nov 30;14(11):e0008793. doi: 10.1371/journal.pntd.0008793 (PMC7728388; doi:10.1371/journal.pntd.0008793)
Supplement: S7 Table — (DOCX) [file pntd.0008793.s009.docx]

**S7 Table.** Mean score by age

| **Age group** | ***m*** | ***N*** | ***SD*** | ***SE*** |
| --- | --- | --- | --- | --- |
| 35 years and less | 62.97 | 91 | 13.864 | 1.453 |
| Above 35 years | 64.70 | 27 | 16.028 | 3.085 |
| Overall average | 63.36 | 118 | 14.335 | 1.320 |

*m*= Mean Knowledge Score *N*=Number of health workers, *SD*= Standard deviation, *SE*= Standard error
