# Supplementary material for: Knowledge of health workers on snakes and snakebite management and treatment seeking behavior of snakebite victims in Bhutan
Source: PLoS Negl Trop Dis. 2020 Nov 30;14(11):e0008793. doi: 10.1371/journal.pntd.0008793 (PMC7728388; doi:10.1371/journal.pntd.0008793)
Supplement: S8 Table — (DOCX) [file pntd.0008793.s010.docx]

**S8 Table.** Mean Score per averaged number of snakebite managed by health workers

| **Number of snakebite managed** | ***m*** | ***N*** | ***SD*** | ***SE*** |
| --- | --- | --- | --- | --- |
| None | 58.65 | 23 | 11.175 | 3.581 |
| Five or less | 62.70 | 47 | 12.723 | 1.856 |
| More than five | 66.27 | 48 | 13.968 | 2.016 |
| Overall average | 63.36 | 118 | 14.335 | 1.320 |

*m*= Mean Knowledge Score, *N*=Number of health workers, *SD*= Standard deviation, *SE*= Standard error
