# Supplementary figures and images for: Knowledge of health workers on snakes and snakebite management and treatment seeking behavior of snakebite victims in Bhutan
Source: PLoS Negl Trop Dis. 2020 Nov 30;14(11):e0008793. doi: 10.1371/journal.pntd.0008793 (PMC7728388; doi:10.1371/journal.pntd.0008793)

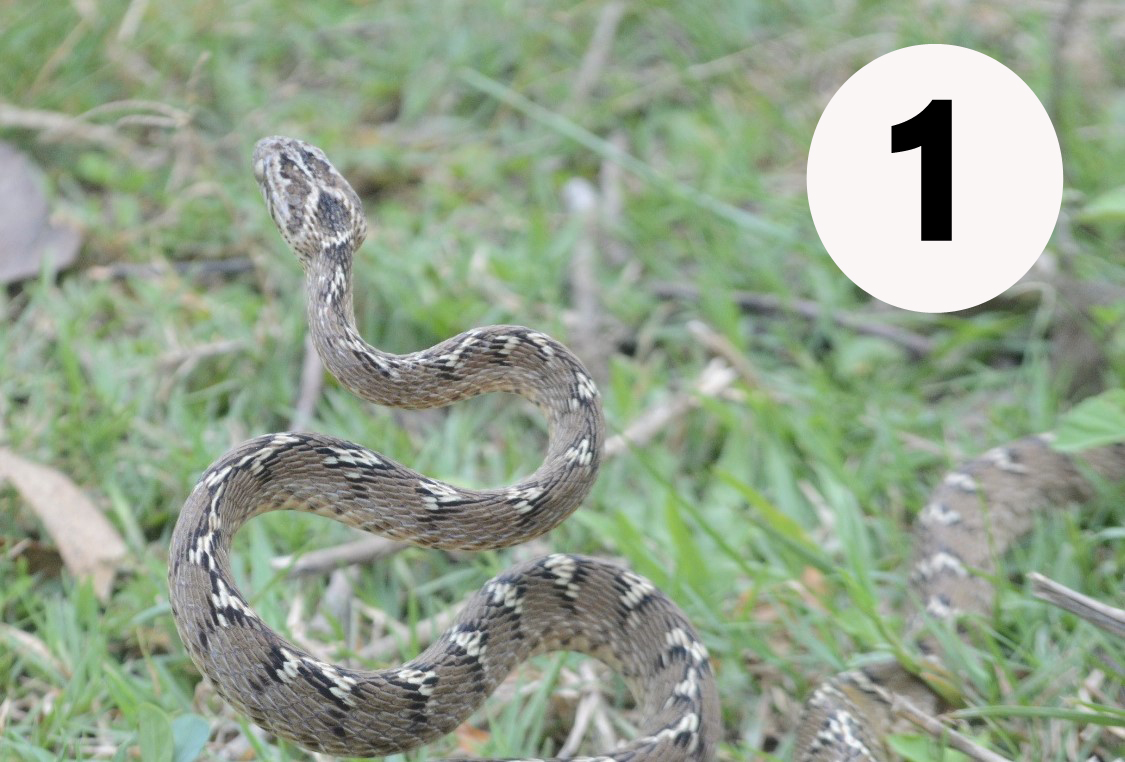

Supplement: S1 Fig — (JPG) [file pntd.0008793.s011.jpg]

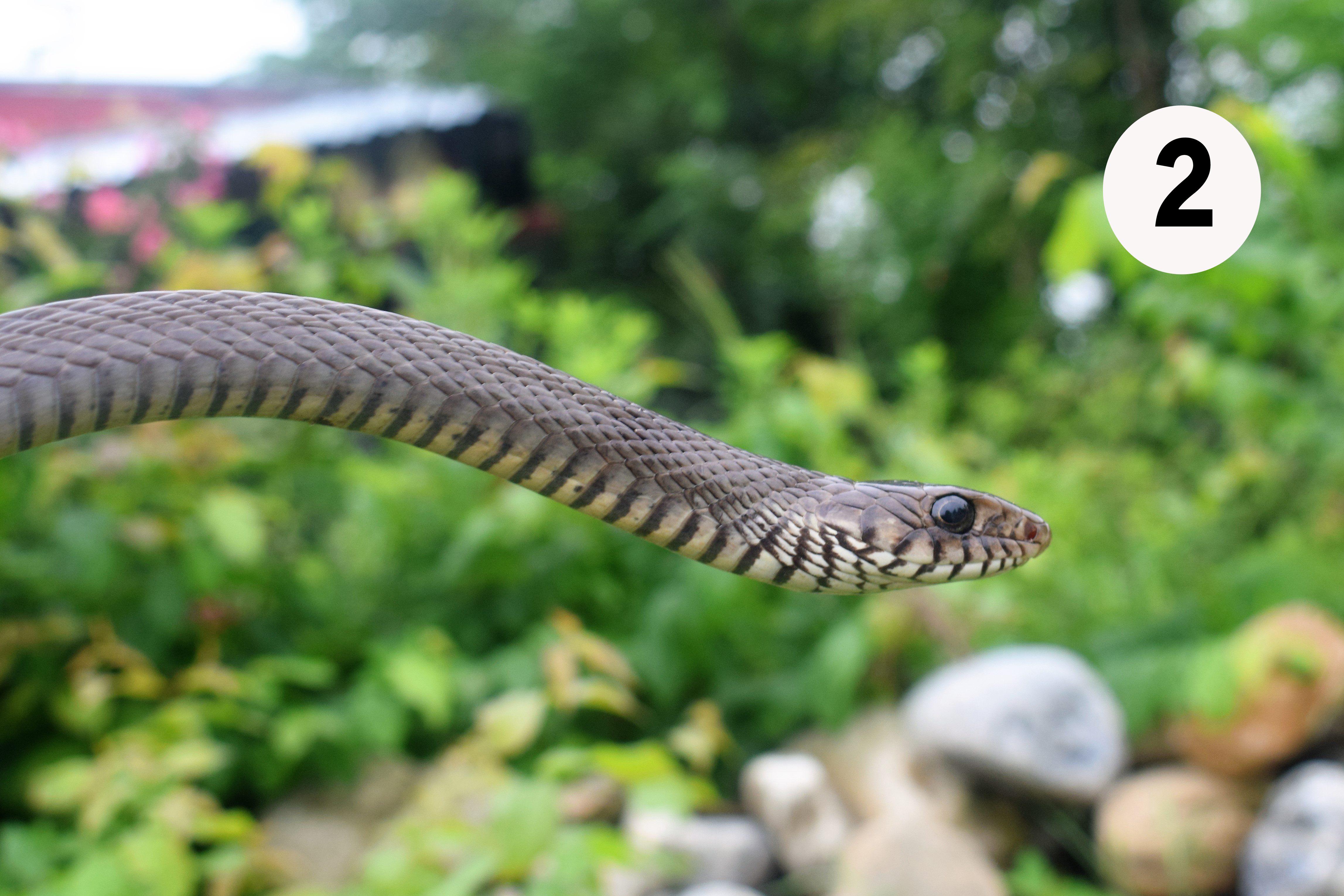

Supplement: S2 Fig — (JPG) [file pntd.0008793.s012.jpg]

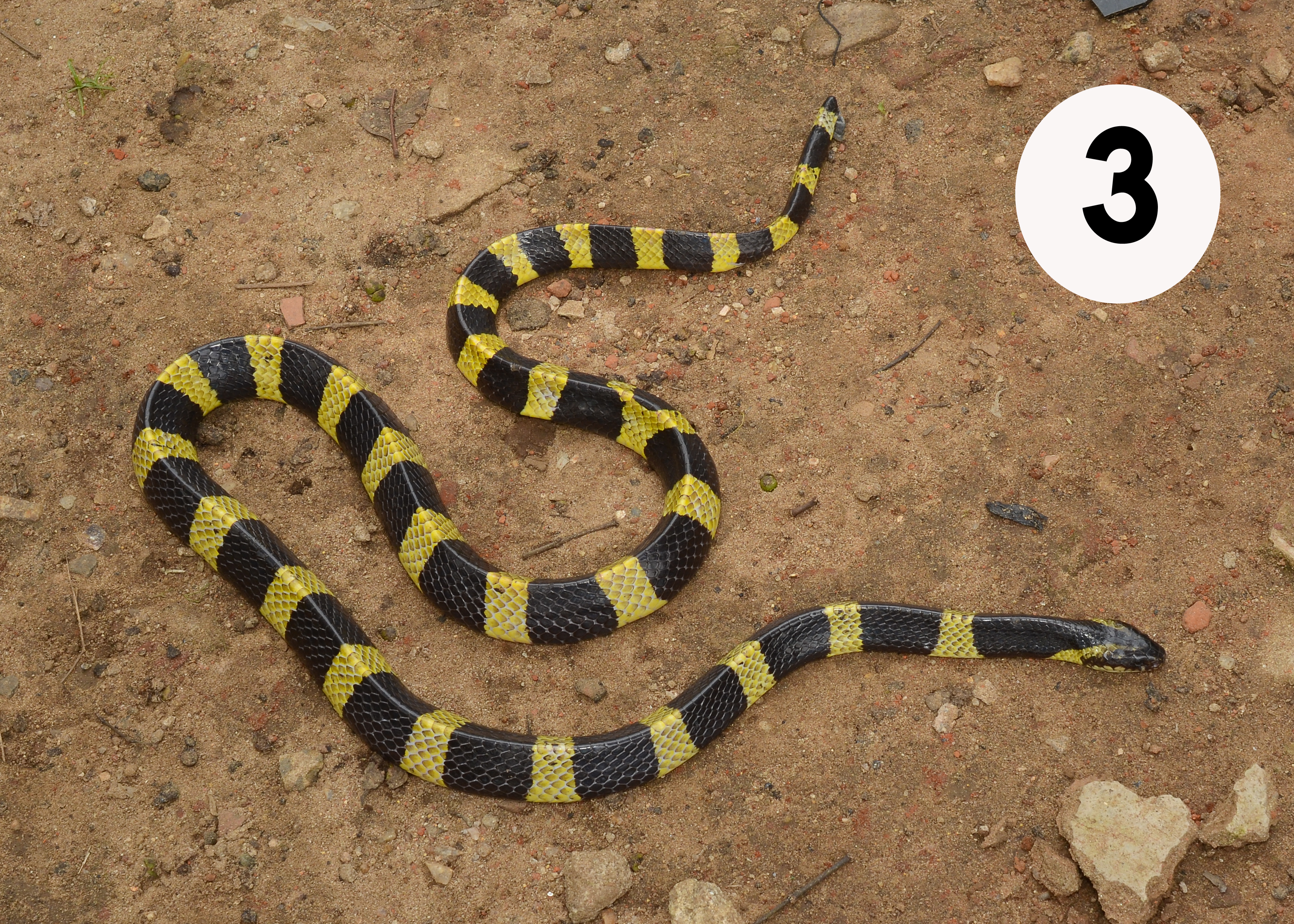

Supplement: S3 Fig — (JPG) [file pntd.0008793.s013.jpg]

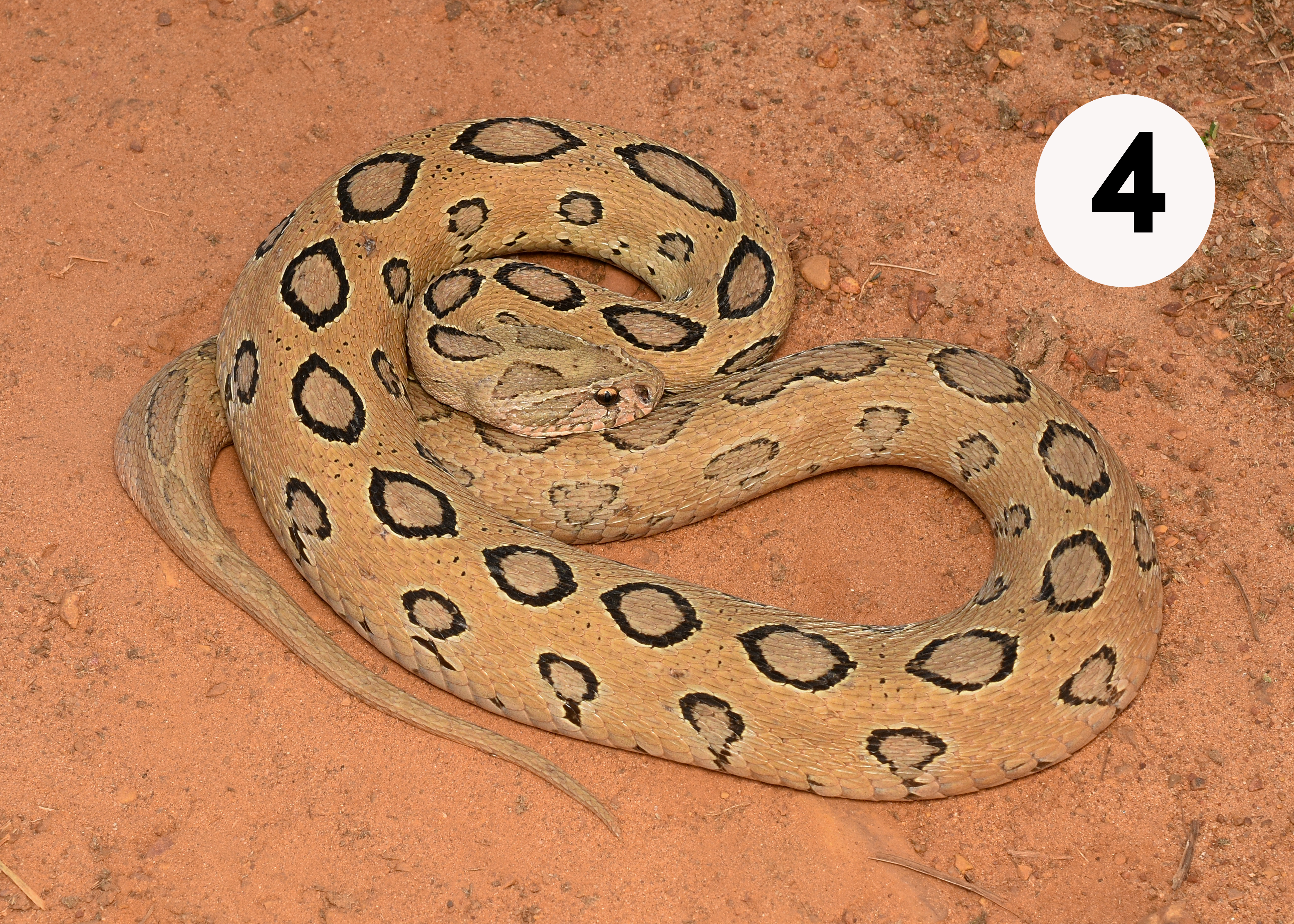

Supplement: S4 Fig — (JPG) [file pntd.0008793.s014.jpg]

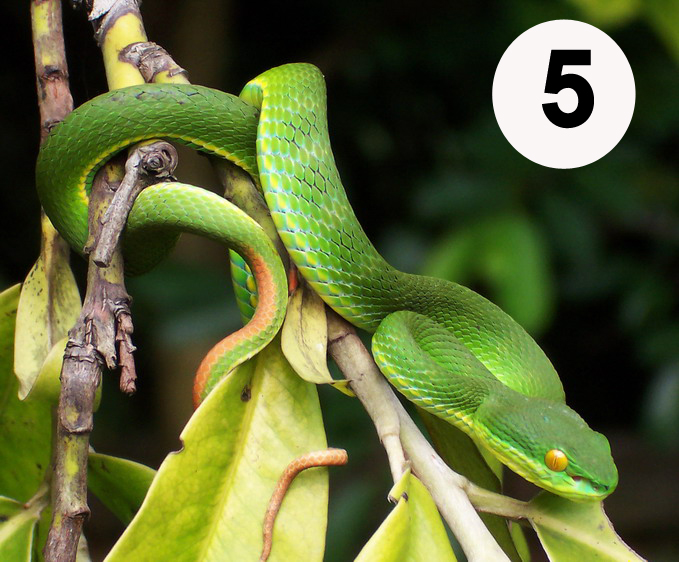

Supplement: S5 Fig — (JPG) [file pntd.0008793.s015.jpg]

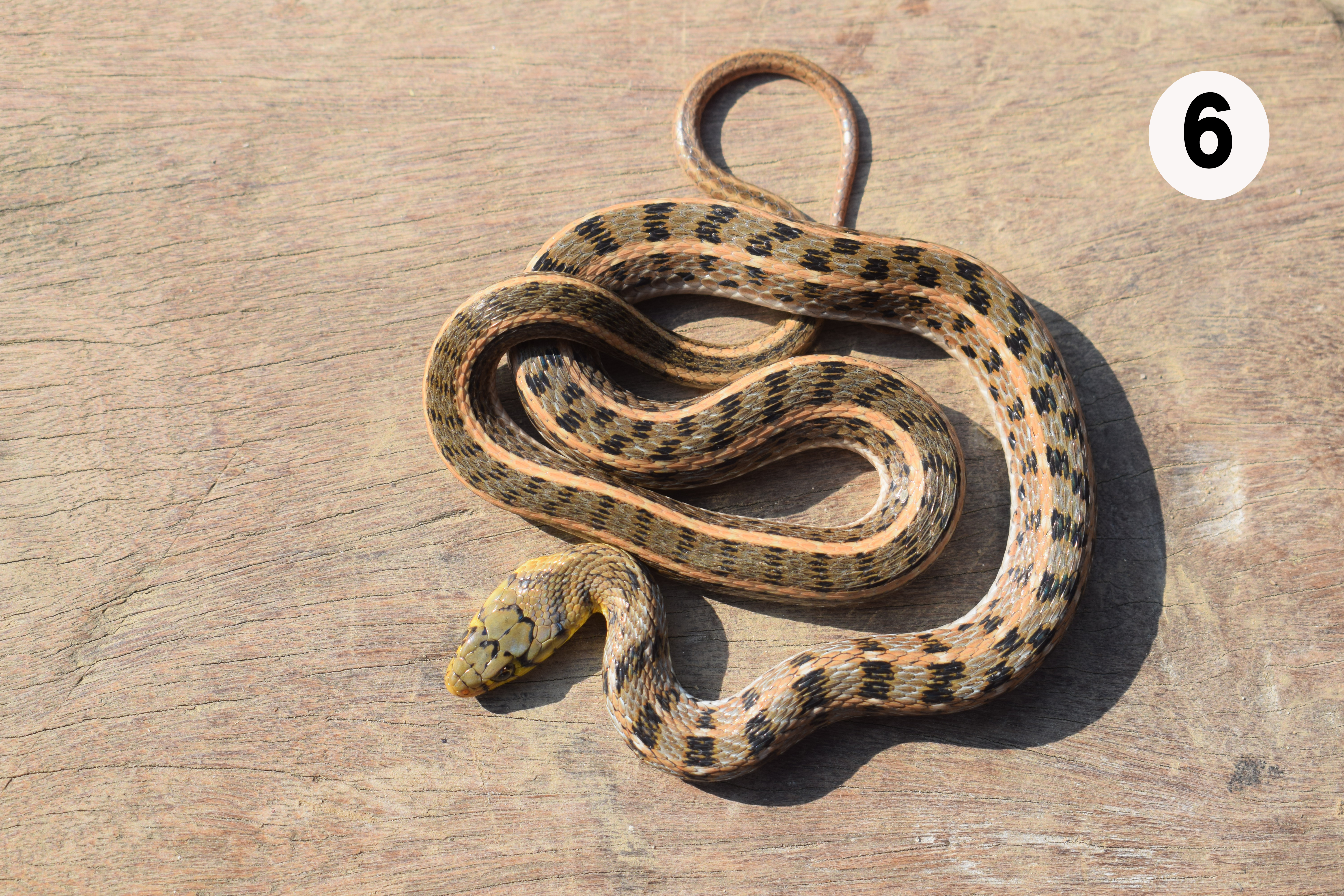

Supplement: S6 Fig — (JPG) [file pntd.0008793.s016.jpg]

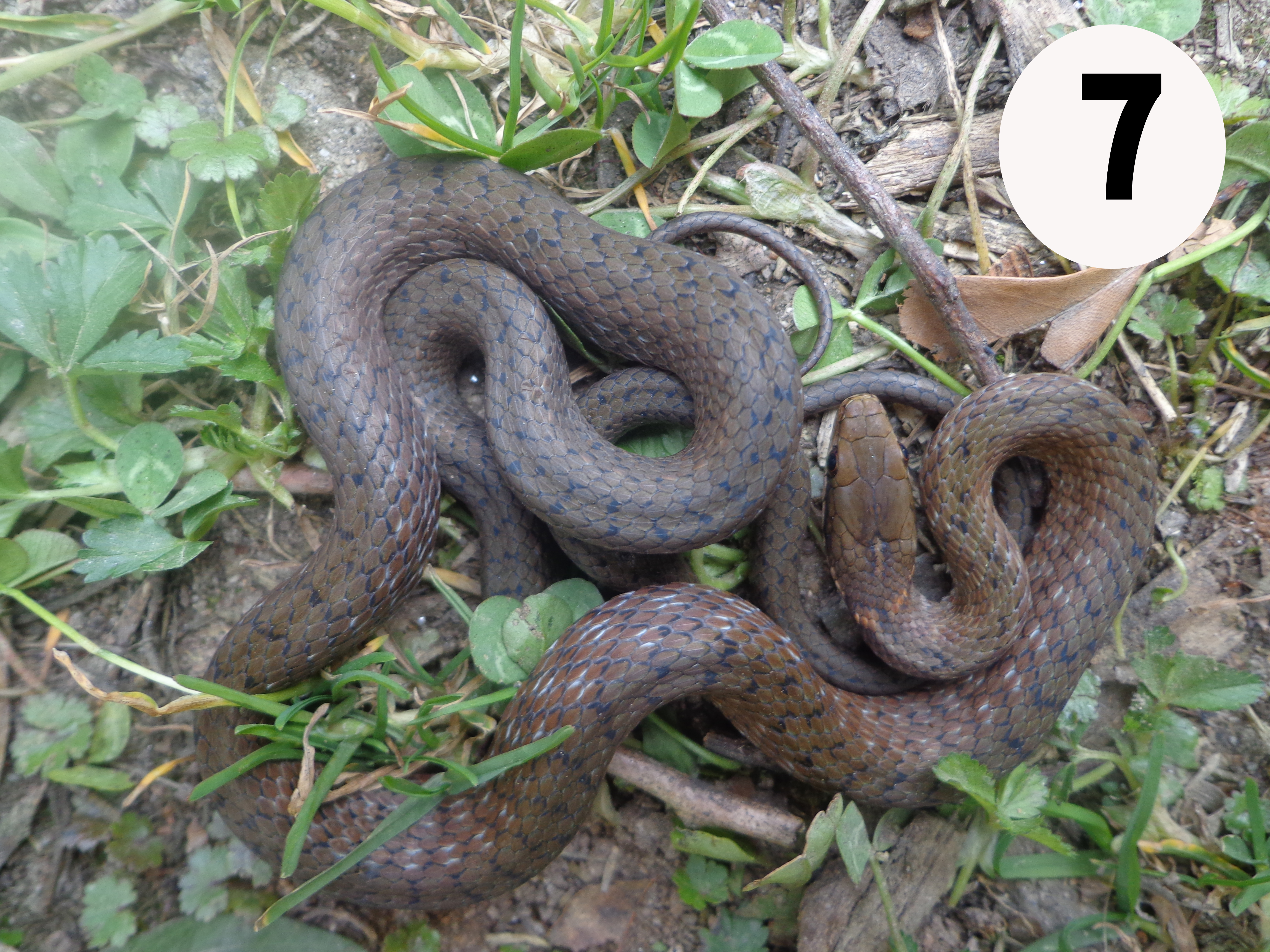

Supplement: S7 Fig — (JPG) [file pntd.0008793.s017.jpg]

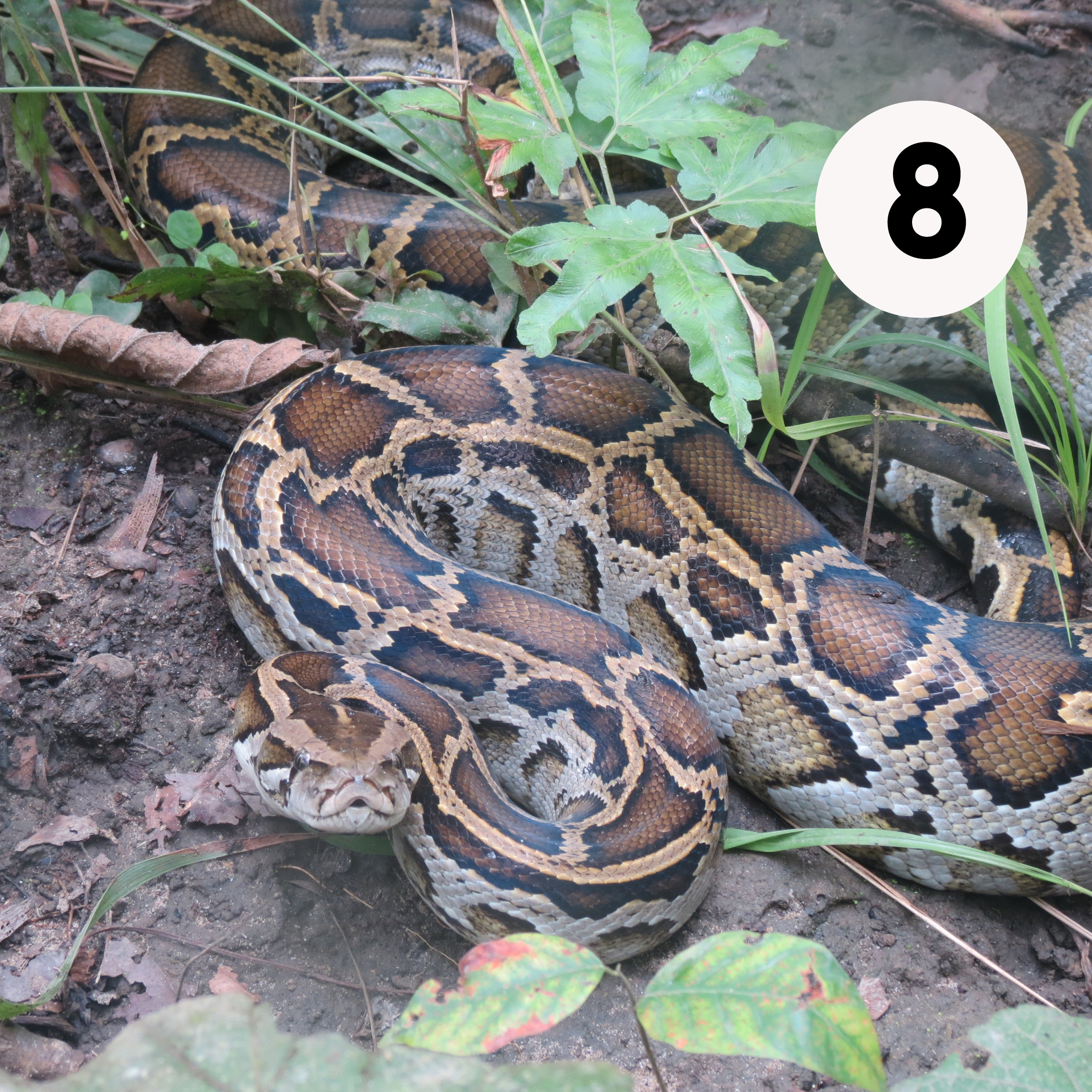

Supplement: S8 Fig — (JPG) [file pntd.0008793.s018.jpg]

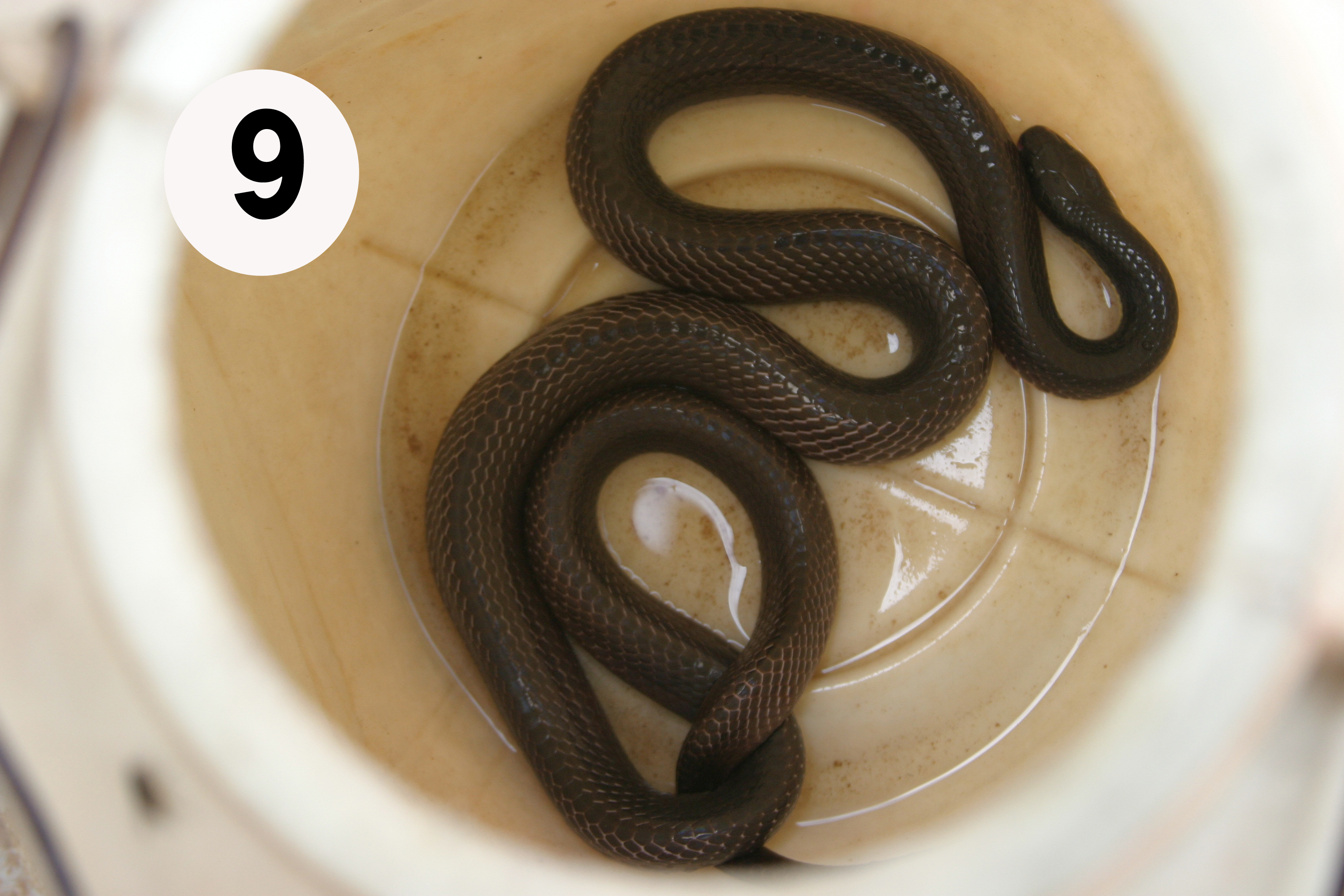

Supplement: S9 Fig — (JPG) [file pntd.0008793.s019.jpg]

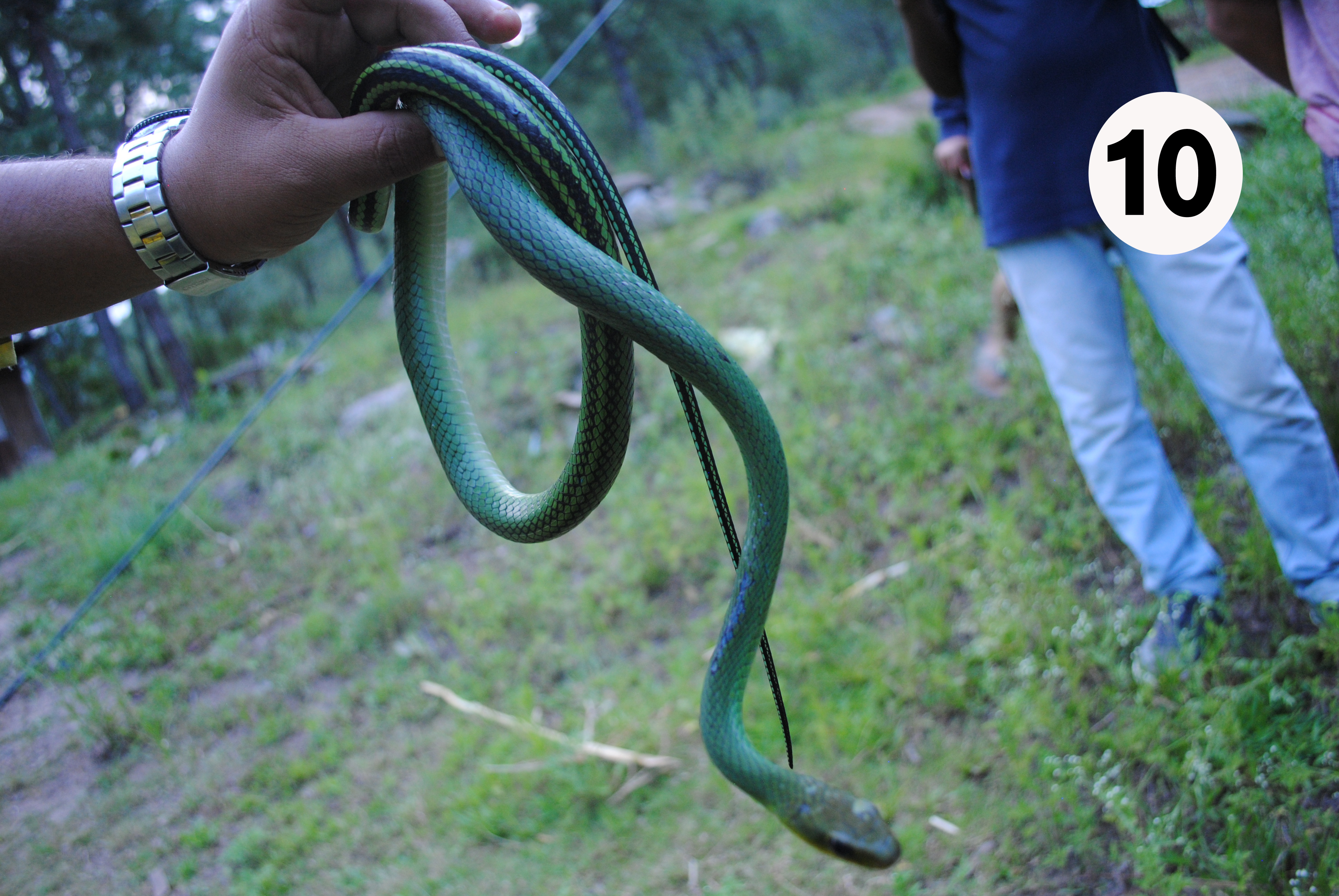

Supplement: S10 Fig — (JPG) [file pntd.0008793.s020.jpg]

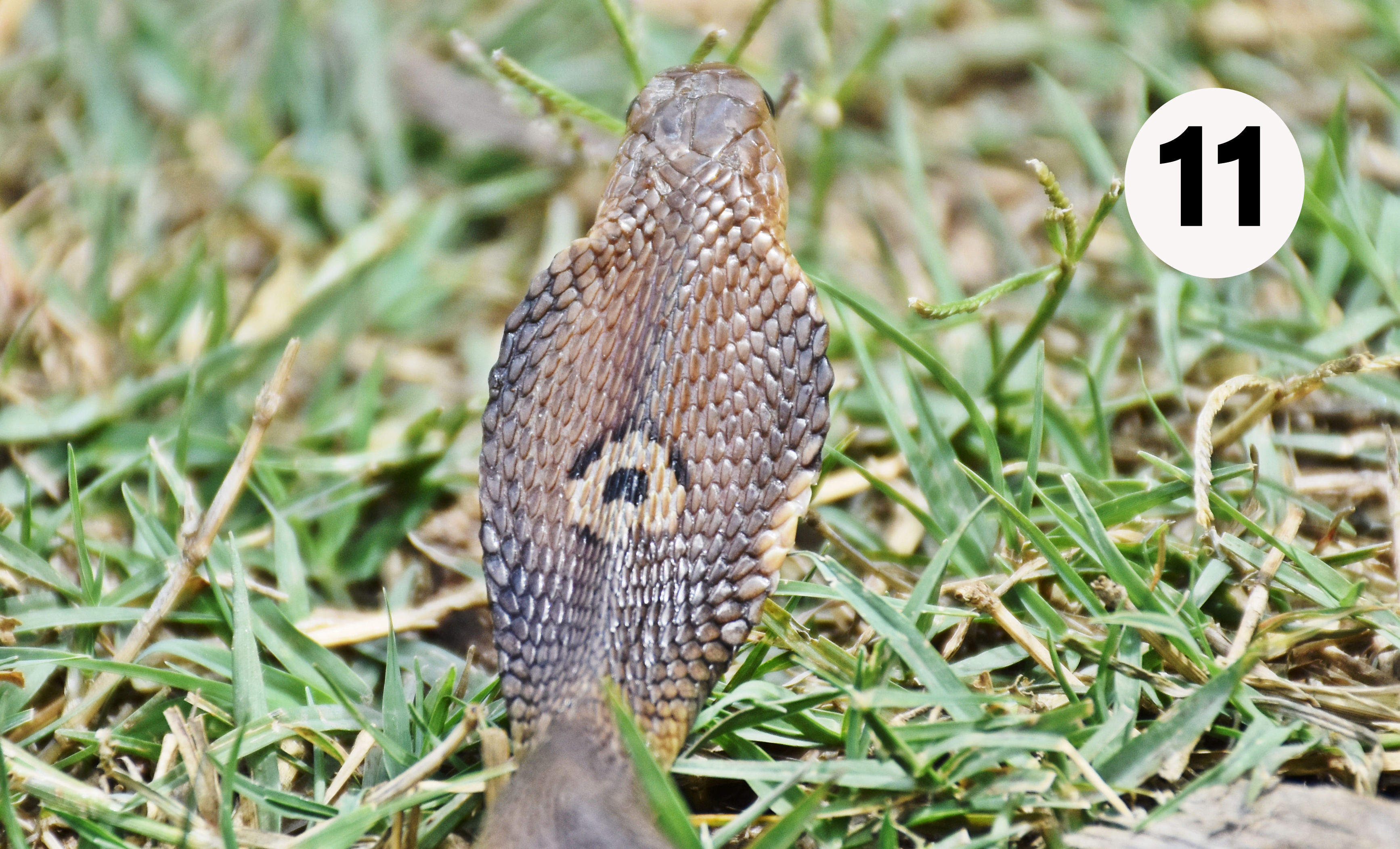

Supplement: S11 Fig — (JPG) [file pntd.0008793.s021.jpg]

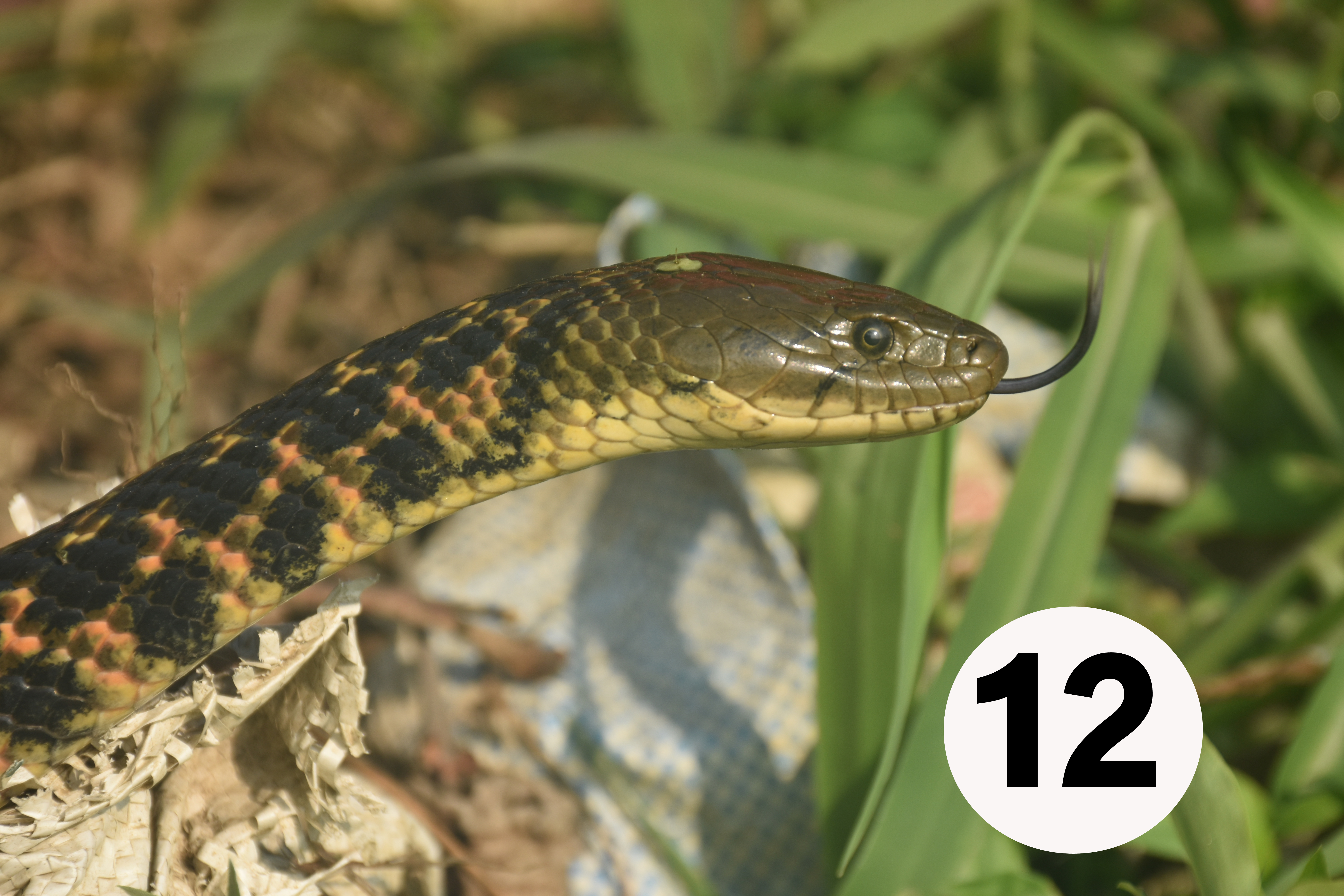

Supplement: S12 Fig — (JPG) [file pntd.0008793.s022.jpg]

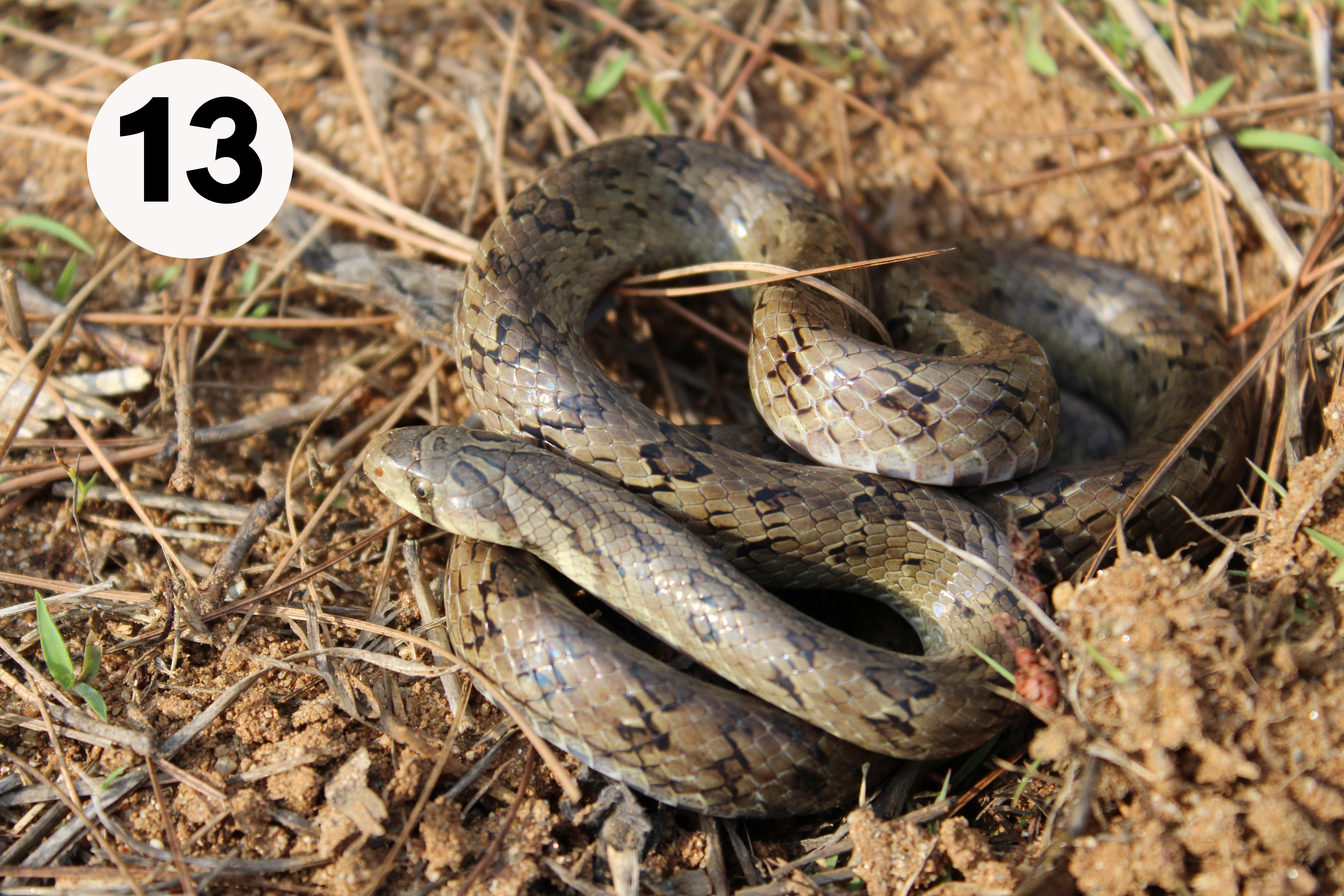

Supplement: S13 Fig — (JPG) [file pntd.0008793.s023.jpg]

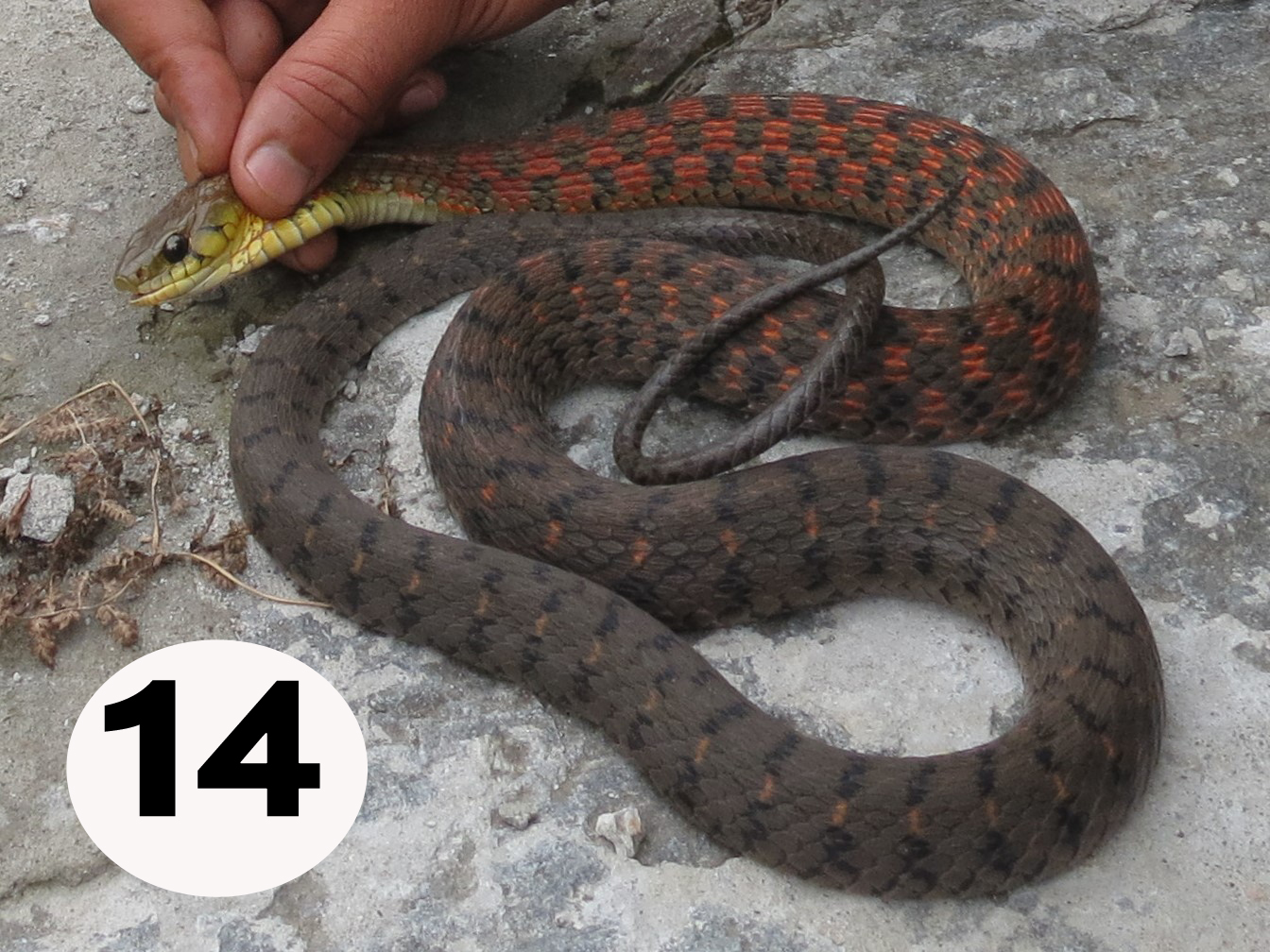

Supplement: S14 Fig — (JPG) [file pntd.0008793.s024.jpg]

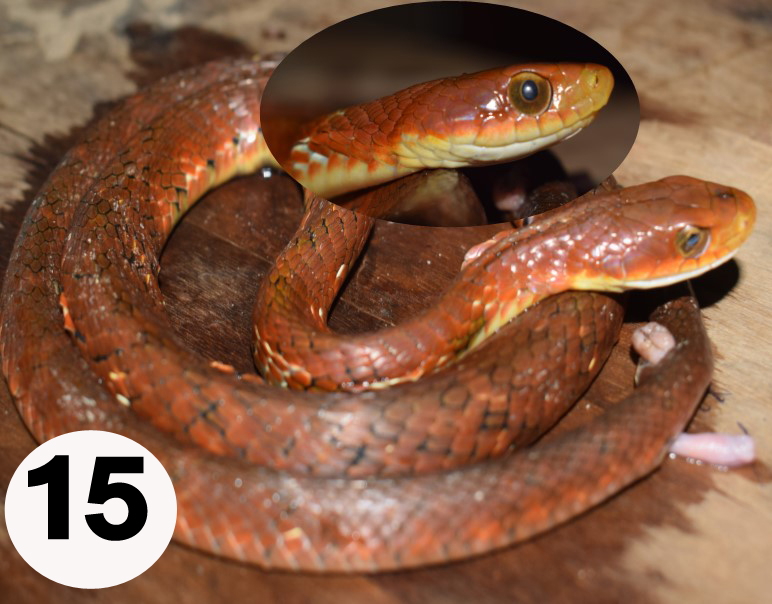

Supplement: S15 Fig — (JPG) [file pntd.0008793.s025.jpg]

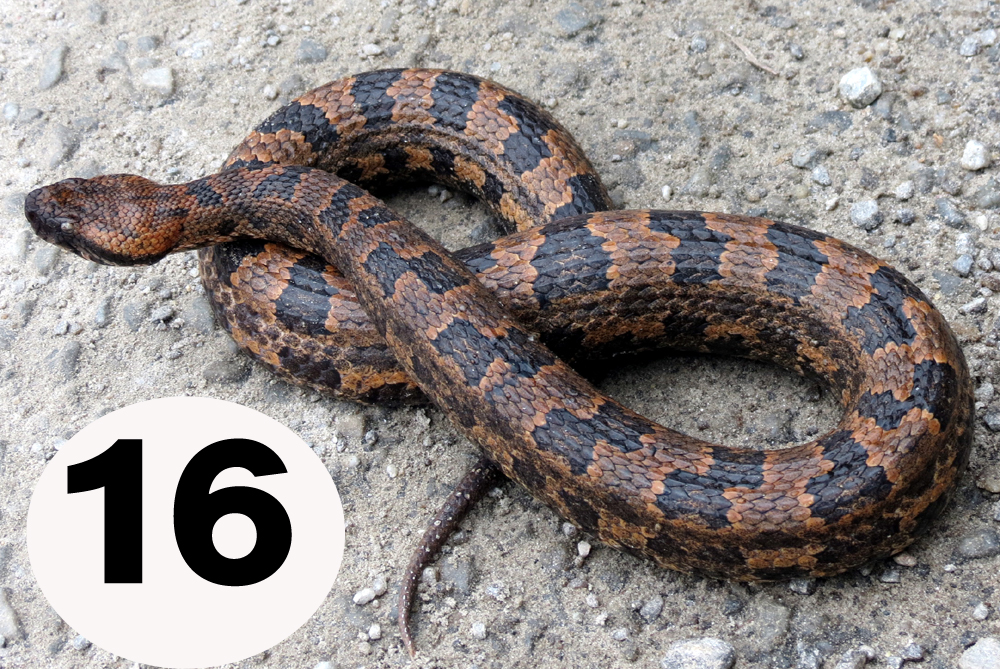

Supplement: S16 Fig — (JPG) [file pntd.0008793.s026.jpg]

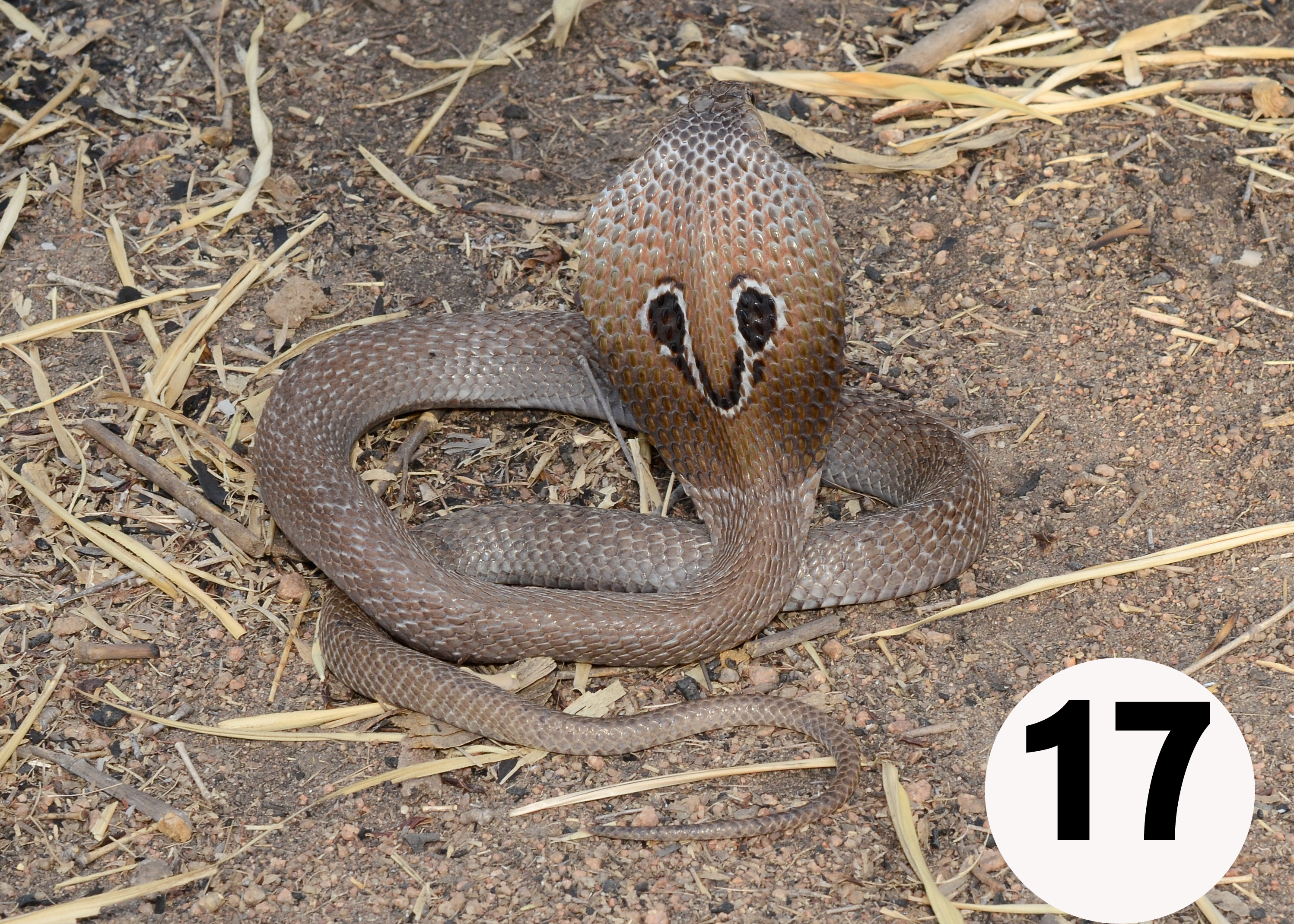

Supplement: S17 Fig — (JPG) [file pntd.0008793.s027.jpg]

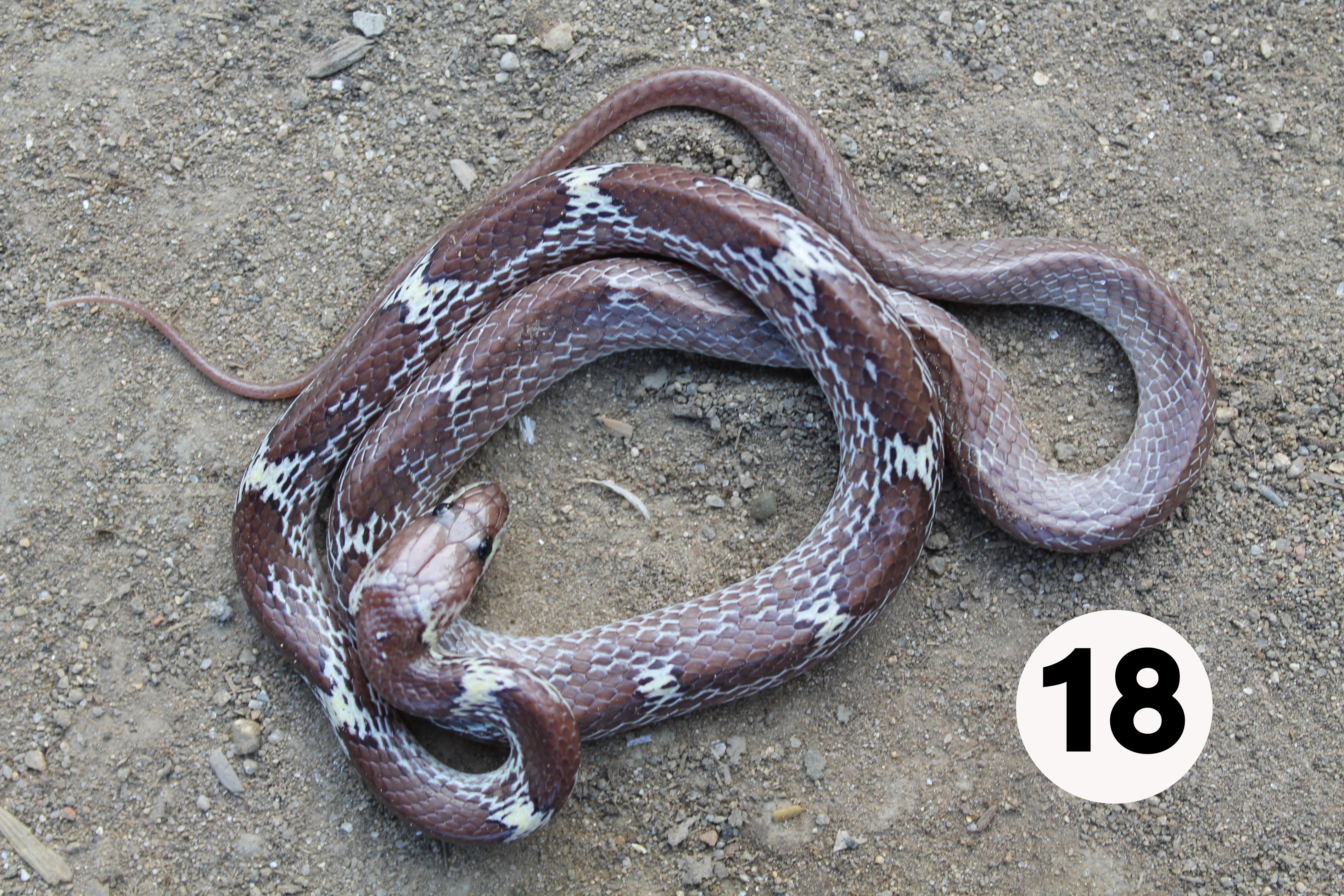

Supplement: S18 Fig — (JPG) [file pntd.0008793.s028.jpg]

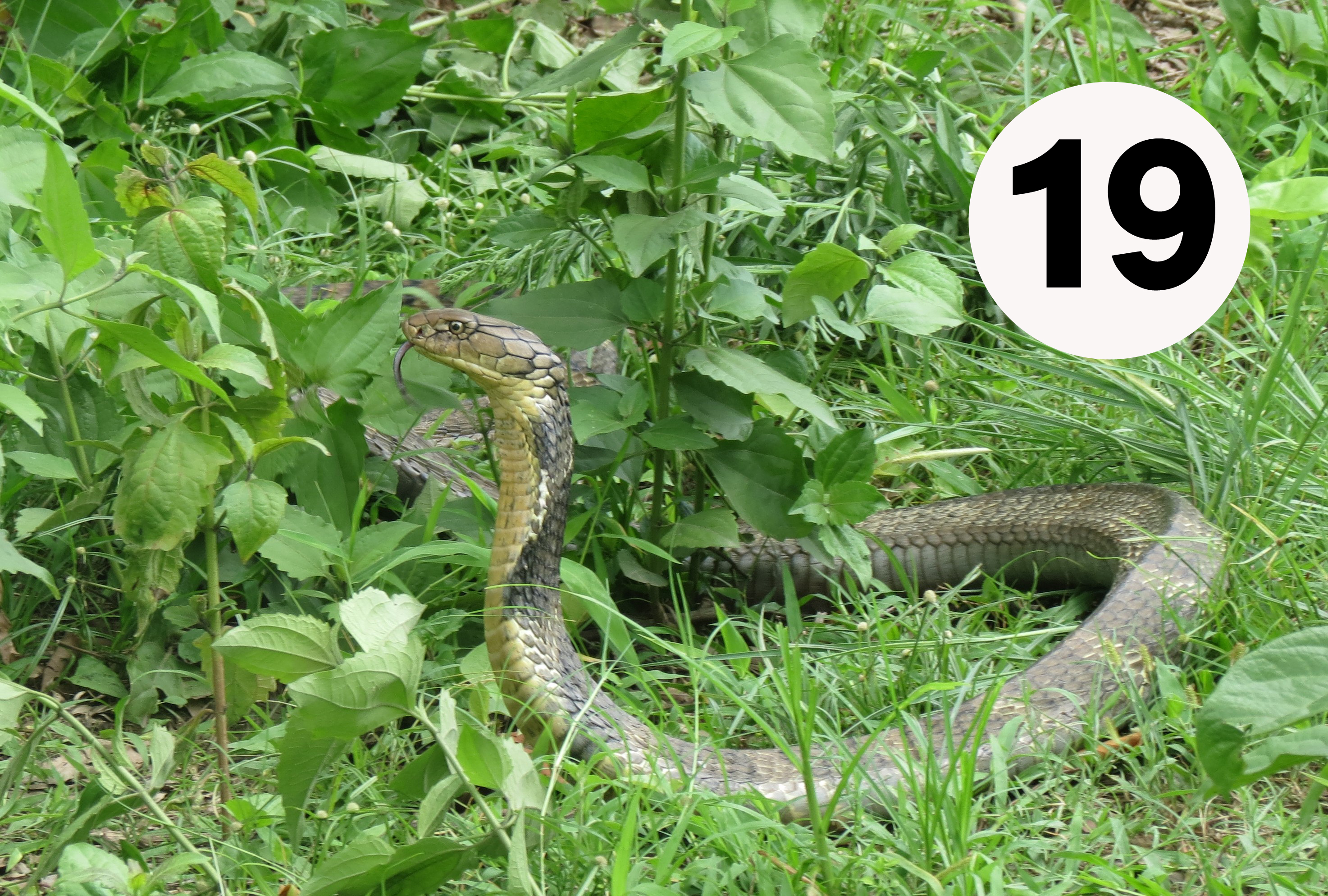

Supplement: S19 Fig — (JPG) [file pntd.0008793.s029.jpg]

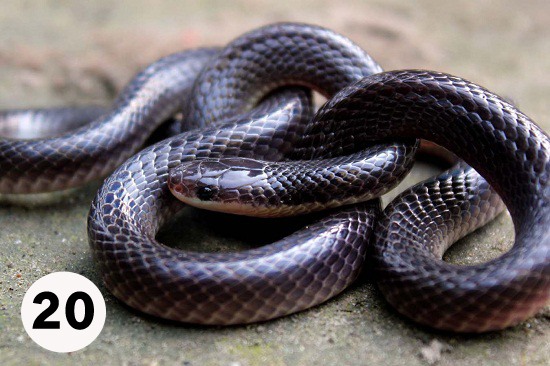

Supplement: S20 Fig — (JPG) [file pntd.0008793.s030.jpg]
